# Targeted suppression of SPP1 inhibits tumor invasion and metastasis in NRF2 hyperactivated cisplatin resistant HNSCC

**DOI:** 10.1186/s12967-026-08292-x

**Published:** 2026-05-22

**Authors:** Mutsuki Kawabe, Sujuan Yang, Lorena I. Gomez-Bolanos, Shiro Takamatsu, Sylvia Flores, Patricia D. Castro, Tsung-You Tsai, Arisa Nishikawa Kaga, Kento Okamoto, Mitchell J. Frederick, Vlad C. Sandulache, Humam Kadara, Jeffrey N. Myers, Abdullah A. Osman

**Affiliations:** 1 Department of Head and Neck Surgery, The University of Texas MD Anderson Cancer Center, 1515 Holcombe Blvd, Houston, TX 77030-4009 USA; 2 Department of Otolaryngology-Head and Neck Surgery, Baylor College of Medicine, Houston, TX USA; 3 Department of Translational Molecular Pathology, The University of Texas MD Anderson Cancer Center, Houston, TX USA; 4 Department of Gynecologic Oncology and Reproductive Medicine, The University of Texas MD Anderson Cancer Center, Houston, TX USA

**Keywords:** Cisplatin, CDDP, Head and neck cancer, Osteopontin, SPP1, KEAP1, NRF2, Integrins, CD44

## Abstract

**Background:**

Cisplatin remains the standard systemic therapy for the definitive treatment of head and neck squamous cell carcinoma (HNSCC), however, resistance to cisplatin continues to be a major barrier to effective treatment, particularly in tumors with NRF2 hyperactivation. Recent studies identify secreted phosphoprotein 1 (SPP1/osteopontin) as a key NRF2 target frequently overexpressed in cancers, where it drives aggressive tumor behavior, metastasis, chemoresistance, and, in some cases, immune suppression. Our recent data highlight SPP1 as one of the top 10 NRF2-upregulated genes in cisplatin-resistant HNSCC. However, its specific role in therapy resistance and metastasis in HNSCC remains unclear. Here, we investigate whether targeting SPP1 can suppress tumor aggressiveness and improve cisplatin response in HNSCC.

**Methods:**

Using established human HNSCC cell lines and mouse models, we utilized conventional western blotting, cell invasion, functional proteomics and high resolution spatial transcriptomics to examine the role of SPP1 in driving tumor progression and metastasis in therapy-resistant HNSCC.

**Results:**

Targeted suppression of SPP1 improved cisplatin sensitivity, inhibited tumor invasion and metastasis both in vitro and in vivo and reduced expression of several metastatic signaling proteins in NRF2-hyperactivated HNSCC. Proteomic analysis revealed that silencing SPP1 led to dysregulation of critical oncogenic and metastatic signaling pathways, including MAPK, AKT/mTOR, FAK, and PAK1. Spatial transcriptomic analysis uncovered a potential mechanistic interaction between SPP1, integrins and CD44 receptors in both primary and metastatic HNSCC. Spatial annotation and enrichment analyses using HALLMARK revealed gene set signatures of interferon and EMT present in cell clusters with SPP1 expression in both the primary tumor and lung metastases. Finally, increased expression of SPP1 was found to be poor prognostic factor and significantly correlated with *NFE2L2/KEAP1* mutational status and higher tumor grade in HNSCC patients.

**Conclusions:**

Targeting dysregulated SPP1 improved cisplatin sensitivity and suppressed tumor invasion and metastasis in NRF2-hyperactivated HNSCC, underscoring the therapeutic potential of SPP1 inhibitors to improve patient outcomes.

**Supplementary Information:**

The online version contains supplementary material available at 10.1186/s12967-026-08292-x.

## Introduction

Head and neck squamous cell carcinoma (HNSCC) is the seventh leading cause of cancer deaths worldwide, with 700,000 cases diagnosed per year [1–3], and the overall 5-year survival remains ~ 50% despite efforts to improve therapeutic regimens [1, 2, 4, 5]. In the recurrent/metastatic setting, HNSCC is nearly universally fatal. Although immune checkpoint inhibitors have been approved for recurrent metastatic HNSCC, only a small subset of patients (< 20%) benefit from this treatment [6–8]. Cisplatin (CDDP) is the gold standard systemic agent for definitive treatment of HNSCC and when combined with immune checkpoint inhibitors (ICIs) for recurrent/metastatic tumors in patients with low combined positive score (CPS > 1) of PD-L1 expression in their tumors [5]. Unfortunately, continuous exposure to cisplatin is frequently associated with substantial toxicity and can result in the development of acquired resistance (both extrinsic and intrinsic), and therefore some HNSCC tumors show lack of response to cisplatin [9–11]. This phenotype has recently been shown by us and others to be driven by hyperactivation of the KEAP1/NRF2 pathway caused by inactivating mutations of *KEAP1* and epigenetic reprogramming of *NFE2L2* (NRF2), resulting in accelerated rates of cervical and lung metastasis in vivo in HNSCC preclinical models [12]. This is further supported by data suggesting that cells developing cisplatin resistance often exhibit a shift towards a more cellular reductive state [11].

Secreted phosphoprotein 1 (SPP1), also known as osteopontin (OPN), is a key NRF2 target gene frequently overexpressed in various cancers and associated with poor prognosis and chemoresistance, particularly in lung, breast, and colorectal tumors [11–14]. Despite high expression in cancer cells and stroma, there is little evidence supporting mechanistic role of SPP1 in tumor progression and metastasis. SPP1 functions as an extracellular matrix soluble oncoprotein through interaction with integrins and CD44 variants and is also involved in cellular migration, invasion, immune evasion, and tumor metastasis [12, 15–18]. Our recently published data identified SPP1 as one of the top 10 NRF2 downstream target genes that was significantly upregulated in vivo in the primary and metastatic tumors in the cisplatin resistant HNSCC cisplatin–treated group compared to the cisplatin-sensitive parental group [10]. However, the role of dysregulated SPP1 in cisplatin-resistant HNSCC and development of distant metastasis has not been fully characterized. The current study aims to investigate whether dysregulated SPP1 promotes tumor progression and metastasis, particularly in the context of Nrf2 hyperactivated cisplatin resistant HNSCC. We leveraged our established head and neck cancer preclinical models, which demonstrated in vitro and in vivo cisplatin resistance that is functionally dependent on dysregulated Nrf2 signaling pathway and associated with high rates of cervical and distant metastasis [10, 11, 19]. In this study we demonstrate that suppressing SPP1 enhances cisplatin sensitivity, inhibits tumor invasion, and reduces metastasis in preclinical models of HNSCC. This effect is linked to ferroptosis-induced cell death rather than apoptosis. Proteomic analysis uncovers dysregulation of key oncogenic and metastatic pathways, including MAPK, AKT/mTOR, FAK, and PAK1, in SPP1-silenced cells. Spatial transcriptomics reveals a potential regulatory axis involving tumor-derived SPP1, integrins, and CD44, which is validated by co-immunoprecipitation analysis. TCGA RNA-seq analysis further associates high SPP1 expression with poor prognosis, NRF2/KEAP1 mutations and aggressive HNSCC features.

In summary, our data suggest that SPP1 promotes tumor progression in NRF2-hyperactivated cisplatin-resistant HNSCC. Selective targeting of dysregulated SPP1 may offer an effective therapeutic approach for treating therapy resistant and metastatic head and neck cancers.

## Methods

### Cell lines and cell culture

HN30 (RRID: CVCL_5525), PCI13 (RRID: CVCL_C182), PCI13 cisplatin-resistant derivatives (PCI13-wtp53, wildtype), HN30-R8, HEK293-FT (ThermoFisher, R70007), were cultured in DMEM (Gibco), containing 10% FBS, l-glutamine, sodium pyruvate, nonessential amino acids, and vitamin solution. All experiments were performed using cells from early passages routinely tested with 0.2% Myco-Zap (Lonza) to ensure mycoplasma-free culture environment [10]. The HNSCC cell lines were authenticated using short tandem repeat analysis [20] within 6 months of use for the current study. The cisplatin-resistant variants HN30-R8, PCI13-wtp53 cell lines were established as previously described [10, 11, 19].

### Generation of SPP1 knockdown stable cell lines

The lentiviral shRNA plasmids, pGIPZ-NT (Cat# RHS4348), pGIPZ-SPP1#1 (Clone # V2LHS_303525), pGIPZ-SPP1#2 (Clone # V2LHS_303526), pGIPZ-SPP1#3 (Clone # V2LHS_303528), pGIPZ-SPP1#4 (V2LHS_111534) were all obtained from Dharmacon. HN30-R8 cells stably expressing the lentiviral non-targeting (NT)control and the pGIPZ-SPP1 plasmids were generated as previously described [10]. Briefly, virus containing supernatants were collected 48 h and 72 h after co-transfection of the pGIPZ-NT, pGIPZ-SPP1 plasmid and the lentiviral packaging vectors pCMV-dR8.2 dvpr and pMD2.G into HEK293-FT cells (using Lipofectamine 2000). HN30- R8 target cells were infected with the virus in the presence of polybrene (polybrene (5 µg/mL) and cells were selected first with puromycin (1.0 µg/mL) followed by sorting of GFP-positive cells. SPP1 knockdown in the stable cell lines were confirmed by Western blotting. The clones showed substantial degree of SPP1 knockdown (Clone #2 and #4) were established and used for further analysis.

### Clonogenic survival and soft agar colony formation assays

To determine colony survival, HN30-R8 (cisplatin resistance) cells stably expressing lentiviral vector control (shCtrl) and SPP1 knockdown derivatives (shRNA SPP1 #2 and SPP1 shRNA #4) were seeded in 6-well plates and exposed to different fixed-ratios of cisplatin (CDDP; 0–20 µmol/L). To block SPP1 binding to integrin receptors, cells were treated with the pan-integrin antagonist GLPG-0187 (0–0.5 µmol/L; MedChemExpress, Cat. No. HY-100506). Clonogenic survival and soft agar assays were performed at these concentrations as previously described by our group [21, 22].

### Western blot analysis

Western blot analysis was performed as previously described [21]. According to the IC50 values, minimally toxic drug doses of cisplatin (CDDP; 5–8 µmol/L) were identified from clonogenic survival assays and used for further in vitro experiments. The HNSCC cells and their genetically modified derivatives were treated in 10-cm dishes with CDDP (8 µmol/L) for 12, 24, and 48 h.

#### Whole cell lysates preparation

Briefly, cells were lysed in RIPA extraction buffer supplemented with a protease and phosphatase inhibitor cocktail. Protein concentrations were determined using the BCA Protein Assay Kit (Bio-Rad Laboratories).

#### SDS-PAGE analysis and ECL detection

Equal amounts of protein were resolved by SDS–PAGE and transferred onto polyvinylidene fluoride (PVDF) membranes (Millipore Sigma). Membranes were blocked with 5% skim milk in TBS-T, incubated overnight at 4 °C with the following antibodies: SPP1 (Cat# 22952-1-AP, Proteintech); NRF2 (Cat# ab137550, Abcam); KEAP1 (Cat# ab119403, Abcam); PARP-1 (Cat #9542), FAK1 (Cat# 13009), phospho-FAK1 (Cat# 8556); all from Cell Signaling Technology; GPX4 (E-12), (Cat# SC-166570), ACSL4 (Cat# SC-271800), all from Santa Cruz Biotechnology; and β-actin (Cat# A2228; Millipore Sigma). Membranes were washed and incubated with the appropriate HRP-conjugated secondary antibodies. Protein bands were detected using an enhanced chemiluminescence (ECL) reagent (VWR International, LLC, Cat#103254-708, CA, USA) and imaged with a ChemiDoc imaging system (Bio-Rad Laboratories). β-actin served as a loading control.

### Immunoprecipitation

#### Whole cell lysates preparation

The whole protein lysates were prepared and collected as described previously in the western blotting method section. The immunoprecipitation was carried out using the Dynabeads™ Co-Immunoprecipitation Kit (Thermo Fisher, Cat#14321D), according to the manufacturer’s instructions.

#### Preparation of the dynabeads

Briefly, the Dynabeads M-270 Epoxy was incubated on a roller at 37 °C overnight with 5 µg antibody per mg beads for antibody coupling. The beads were washed consecutively with the washing buffer and RIPA lysis buffer.

#### Immunocomplexes

The protein lysates were incubated on a roller at 4 °C for 30 min with the coupled beads. The beads were then washed with elution buffer for 5 min. The protein lysates coupled with beads were then incubated with the appropriate antibodies overnight at 4 °C with constant rotation. The beads were separated magnetically, and samples were washed 3 times with the elution buffer followed by SDS-PAGE and Western blotting analyses as described previously [21]. Antibodies used for immunoprecipitation are CD44 (Cat# 3570); from Cell Signaling Technology, Integrin αV/ITGAV/CD51 (P2W7) (Cat# SC-9969), Integrin β1/ITGB1 (A-4) (Cat# SC-374429), Integrin β6 (C-19) (Cat# SC-6632), and Integrin β3/ITGB3/CD61 (D-11) (Cat# SC-365679); all from Santa Cruz Biotechnology.

### Cell invasion assay

Cells were treated with cisplatin (CDDP; 8 µmol/L) and, where indicated, with the pan-integrin antagonist GLPG-0187 (0.25 or 0.5 µmol/L) or a neutralizing antibody cocktail targeting CD44 and integrins α3, α4, αv, and β1. Antibodies were used at a concentration of 15 µg/mL each. Following 24 h of treatment, cells were harvested, resuspended in serum-free medium, and seeded into the upper chamber of Matrigel-coated transwell inserts (Corning, Cat. #354480). Medium supplemented with 10% fetal bovine serum (FBS) was added to the lower chamber as a chemoattractant. After 36 h of incubation, non-migrated cells remaining on the upper surface of the chamber of Matrigel-coated transwell inserts were gently removed using a cotton swab. The cells migrated or invaded through the inserts were fixed with 20% methanol and stained with Hemacolor (Sigma). Stained cells were imaged and quantified using a Leica DMLA microscope (Leica Microsystems). Experiments were performed in duplicate or triplicate and repeated three independent times.

### Lipid peroxidation assay

Lipid peroxidation level in ferroptotic cells was measured using BODIPY 581/59 C11 probe (Invitrogen™, D3861) in accordance with the manufacturer’s instructions. Briefly, the cells were plated in 6-cm dishes and treated with CDDP (8 µmol/l) for 48 h as indicated. After a quick wash with PBS, the cells were incubated with 2 µmol/l of BODIPY and kept in the dark for 30 min at 37 °C. The cells were then rinsed once with PBS to remove the staining solution, trypsinzed, and a minimum of 100,000 cells per dish were analyzed by FACS. Ferrostain-1 (1.0 µmol/L; Cat#HY-100579, MedChemExpress) was used to confirm specific cell death through ferroptosis. Experiments were conducted in triplicate dishes and repeated three independent times.

### Annexin V-APC staining

HNSCC cells were seeded in 60-mm dishes, treated the next day with CDDP (8 µmol/L) and then harvested at 48 h. Apoptosis was detected by flow cytometry using the Annexin V-APC/7-AAD staining kit obtained from BD Bioscience according to the manufacturer’s instructions. The experiment was carried out in triplicate and repeated three independent times.

### TCGA database analysis

Homogenized RSEM count data for the HNSCC TCGA was downloaded from the TCGA TOIL database as we previously described [23] and upper quartile normalized to generate log2 FPKM gene expression with global median rescaling using our recently published RNA-seq pipeline [24]. Clinical annotation and matching mutation data, including status of NRF2 and KEAP1 genes, were downloaded from the BROAD firehose web portal. Recursive partitioning to identify optimal SPP1 RNA thresholds for splitting the oral cavity squamous cell carcinoma (OCSCC) and laryngeal/hypopharyngeal squamous cell carcinoma (LHSCC) cohorts into survival groups was performed with an in-house python script that uses an iterative approach, balances groups, and maximizes significance for both Log-Rank and Gehan-Breslow-Wilcoxon approaches. Differences in SPP1 expression among groups was analyzed by a two-sided student t-test (for 2 groups), or ANOVA (for > 2 groups) with a post-hoc Tukey’s Honestly Significant Difference test. Differences in proportions of patients based on SPP1 expression group and extracapsular spread was examined with a Chi-squared test. A multiple linear regression model was fit using JMPv13 (SAS) statistical software to determine the dependence of SPP1 expression on NRF2, macrophage, and cancer associated fibroblast (CAF) single sample gene set enrichment scores (ssGSEA) and calculate standardized regression coefficients (beta values), along with associated P-values. The gene set list for the Nrf2 score was developed and validated previously by our group [23], while the macrophage and cancer associated gene lists were developed by vetting and refining previously published lists [25] using their cross-correlation coefficients of expression across more than 9,000 TCGA solid tumors as we previously described [23]. For the multivariate regression, the β values are the standardized regression coefficients in the model to demonstrate relative contributions and statistical significance of the main factors is indicated by the asterisks above the regression coefficients. The BROAD Gene Pattern web server tool was used to calculate individual ssGSEA values from each gene list.

### Patient samples

Following approval from the Baylor College of Medicine Institutional Review Board, archival primary and metastatic tumors from patients with a diagnosis of oropharyngeal squamous cell carcinoma, treated with curative intent at the Michael E. DeBakey Veterans Affairs Medical Center were analyzed. Patient sample characteristics are as follows: Patient #1: T3N2cM0 p16 + OPSCC treated with surgery + radiation + cisplatin, Patient #2: T2N2bM0 p16 + OPSCC treated with radiation + cetuximab, Patient #3: T2N2bM0 p16 + OPSCC treated with radiation + cisplatin, Patient #4: T2N3bM0 p16-OPSCC treated with surgery + radiation + cetuximab, and Patient #5. T4N2bM0 p16 + OPSCC treated with radiation + cisplatin (staging reflects the 7th Edition of the AJCC Staging Manual). Plasma samples for an additional 28 HNSCC patients were analyzed for SPP1 levels using standard ELISA. Patient, site and treatment characteristics for these 28 patients are summarized in Supplementary Table S8. Despite the limited sample size, there was no attrition; all 28 patient samples were successfully analyzed. Because this was a pilot study, a formal power calculation was not performed.

### SPP1 enzyme-linked immunosorbent assay (ELISA)

For detection of SPP1 levels in vitro, cells (1 × 10^6^) were seeded in 100-mm dishes containing complete medium and incubated for 24 h as indicated. The cells were harvested, and the supernatants were prepared from triplicates. To measure the SPP1 in vivo, plasma samples from mice treated with CDDP plus anti-OPN and advanced HNSCC patients treated at Baylor College of Medicine were also obtained. The SPP1 levels were measured using the ELISA kit according to the manufacturer’s instructions (QuantikineTM, Techne Corporation R&D Systems). Briefly, cell supernatants and the plasma samples were added to the appropriate ELISA plates precoated with monoclonal antibodies directed against human OPN (SPP1). After incubation for 2 h at 37 °C, the wells in the plates were washed, and HRP-conjugated polyclonal OPN specific antibody was added. The plates were incubated for 1 h at 37 °C, washed, and followed by addition of tetramethylbenzidine substrate. Finally, the enzymatic reactions were stopped by addition of 2 N sulfuric acid and the results were detected on a microplate reader at 450 nm. Experiments were performed in triplicate and repeated twice (*N* = 2).

### Proteomics

HN30-R8 cells stably expressing lentiviral vector control (shCtrl) and SPP1 knockdown derivative (SPP1 shRNA #4) were seeded in 10-cm dishes in triplicates and harvested at 24 h with RIPA lysis buffer containing protease and phosphatase inhibitors as indicated. Protein lysates were then collected, vortexed, centrifuged and total protein resulting from supernatant for each sample was quantitated using a BCA kit (Pierce Biotechnology Inc., Rockford, IL). Lysates were prepared in biological triplicate and subjected to Reverse phase protein array (RPPA) as described previously [26]. The RPPA was performed by the RPPA core facility at the University of Texas MD Anderson Cancer Center. Protein expression data were generated for 77 protein analytes. RPPA slides were quantified using ArrayPro (Media Cybernetics) to generate signal intensities that were further processed by SuperCurve to estimate relative protein levels (in log2 scale). RPPA samples quality was monitored by a QC classifier and only the slides whose QC scores were above 0.8 (on a 0–1 scale) were used for further analysis. Differences in group mean RPPA analytes were identified with independent two-sample (two-sided) t-tests using JMP13 software, adjusting for multiple comparisons with the Benjamini-Hochberg procedure (q ≤ 0.1). Volcano plots of significance verses differences in RPPA level were generated with GraphPad Prism software, and two-way Ward’s hierarchical clustering for select analytes with the corresponding heatmap was generated in JMP. Significant differentially expressed proteins and phosphoproteins among the groups were subjected to Gene Ontology (GO) enrichment analysis to identify cell function.

### Visium spatial transcriptomics

Archival primary and metastatic FFPE tissue samples from the five recurrent/metastatic head neck cancer patients indicated previously in this study were processed for Visium data pre-processing and quality control to ensure high-quality data and RNA integrity. Briefly, FFPE tissue sections of 5 μm thick were stained with H&E and scanned at 40X using Aperio AT2^®^ scanner (Leica Biosystem). The H&E-stained slides underwent digital histopathology assessment using Image Scope Software X64 for pathology quality control, which included the evaluation of optimal tissue sectioning (absence of folds, tears or detachment), staining intensity and homogeneity, and scanning resolution. Only one patient (T3N2cM0 p16 + OPSCC treated with surgery + radiation + cisplatin) demonstrated optimal pathological quality and was considered for the Visium spatial transcriptomic analysis. The H&E stained slide was subjected for ST tissue processing using Visium CytAssist instrument from 10X Genomics according to the manufacturer protocol and followed by ScRNA-seq sequencing on Illumina iSeq100 for cell count validation and NovaSeq6000 at the recommended depth. To map the whole transcriptome in FFPE tissue, the reads were pre-processed using Space Ranger (v.2.1.0) from 10x Genomics and aligned to the GRCh38 reference genome. The unique molecular identifier (UMI) count matrices were analyzed using Seurat v5 in the R platform (v.4.3.0). Pathology annotations of the tissue histological morphology were performed manually by an experienced pathologist using Loupe Browser 8.0.0with the corresponding high-resolution image. Spots recognized as “excluded” which represented no tissue or damaged tissue, were filtered out during this processing step. For Visium clustering analysis, the SCTransform functions of Seurat was used to normalize and scale the matrix to identify highly variable genes (HVGs). Top 3,000 highly variable genes (HVGs) were selected for Principal Component Analysis (PCA) and downstream unsupervised spot clustering. Differentially expressed genes among clusters were identified with logFC greater than 0.25 (adjusted *p* < 0.05) as determined in Wilcoxon rank-sum test from Seurat. To identify spatially distinct domains, the ‘FindNeighbors’ function of Seurat was used to construct the Shared Nearest Neighbor (SNN) Graph, based on unsupervised clustering performed with ‘FindClusters’ function with a resolution set to 0.5. Subsequently, 2-D visualization of the spot clusters were performed using Uniform Manifold Approximation and Projection (UMAP) with of Seurat function RunUMAP. The top 50 PCs were used to calculate the embedding. The COMMOT (COMMunication analysis by Optimal Transport) tool was used to infer cell–cell communication analysis in spatial transcriptomics. It was installed and imported by Python following developer’s instruction. This tool which contains 1,939 validated molecular interactions and allows for detection of the interaction between different ligand and receptor species as well as spatial distances between cells [27]. The analysis was performed using the standard pipeline shared by the developer. “CellChat” ligand-receptor database was used for “ct.tl.spatial communication”. The gene expression with manual masks was finally visualized using the iSTAR integrative approach which combines spatial transcriptomics with histological features [28]. It is a method based on hierarchical image feature extraction that integrates ST data and high-resolution histology images to predict spatial gene expression with super-resolution. The differential gene expression in tumor cells annotated as squamous cell carcinoma by iSTAR and assigned to clusters 0, 1, 2, 7, 9, and 10 (primary tumor) or clusters 3 and 6 (metastatic lung tumor) were analyzed using the FindMarkers function in Seurat. Genes expressed in fewer than 5% of cells (p < 0.05) were excluded. An adjusted p-value < 0.01 and a log fold change > 1.5 were considered for significantly expressed genes. Gene set enrichment analyses (GESA) of these differentially expressed genes were performed using EnrichR with the 50 MSigDB HALLMARK gene sets.

### Orthotopic nude mouse model of oral cancer metastasis and therapy

All animal experimentation was approved by the Institutional Animal Care and use Committee of the University of Texas MD Anderson Cancer Center. Our orthotopic nude mouse tongue model was previously described in the literature [10]. To determine in vivo growth kinetics and response to cisplatin treatment of HN30-R8 cells with SPP1 knockdown (SPP1 shRNA #2 and #4) and shCtrl vector, cells (5–10 × 10^4^) were suspended in 30 µl of medium without supplement and injected into the lateral tongues of six-week-old male athymic nude mice because oral cancer is mostly prevalent in male patients. Mice were then randomized into different groups 8 to 10 days after injection. Treatment with vehicle (PBS) or CDDP (4 mg/kg, I.V., via tail-vein/once a week for 4 weeks) was started when tumors reached a range of 3 to 6 mm^3^ in size. For the in vivo drug combination experiment, the HN30-R8 CDDP-resistant cells were injected into the mice oral tongues as described above, and treated with either CDDP alone, the SPP1 inhibitor (osteopontin monoclonal antibody, BioXCell #Cat #BE0382, 10 mg/kg, 3 times a week, given intraperitoneally, I.P.) alone, and their combination or PBS vehicle (given I.P.) for 4 weeks. A total of 9–12 mice in each group were used, and 90% of the mice had tumor growth under each condition. Mice were monitored twice a week, and their weight and tumor volume were recorded. Tongue tumors were measured in millimeters with microcalipers by investigator blinded to the treatment groups, and tumor volume was calculated as A × B2 × (π/6), where A is the longest dimension of the tumor and B is the dimension of the tumor perpendicular to A. Mice were euthanized when they lost more than 20% of their preinjection body weight. During necropsy, tongues, cervical lymph nodes and lungs were harvested, formalin-fixed, and subjected to histologic evaluation using hematoxylin and eosin (H&E) staining to identify primary tumors and count the number and size of spontaneous metastases. A separate group of mice were injected with HN30-R8 cells expressing shCtrl vector and SPP1 shRNA#2 via tail vein injection to confirm incidence of spontaneous metastasis. Lungs were harvested 60 days after tail injection of the cells, processed and evaluated with H&E staining as described above.

### Statistical analyses

The student’s t and two-way ANOVA tests were utilized to analyze in vitro and in vivo data. Mice survival following tumor formation or drug treatment was analyzed by the Kaplan–Meier method and compared with log-rank test. Student t-test statistic value of *P* < 0.05 was used to compare ELISA results in mice and human plasma samples. All data were expressed as mean ± standard error, and cutoff p-values of 0.05 or less were considered to indicate statistical significance. In vitro experiments were carried out in triplicate or duplicate as appropriate (for each condition) and were repeated to ensure reproducibility.

### Study approval

All animal studies contained herein were conducted following approval by, and in accordance with the rules of the MD Anderson Cancer Center Institutional Animal Care and Use Committee.

## Results

### SPP1 targeted suppression enhances cisplatin sensitivity in vitro in cisplatin resistant HNSCC through induction of ferroptosis

**Fig. 1:**
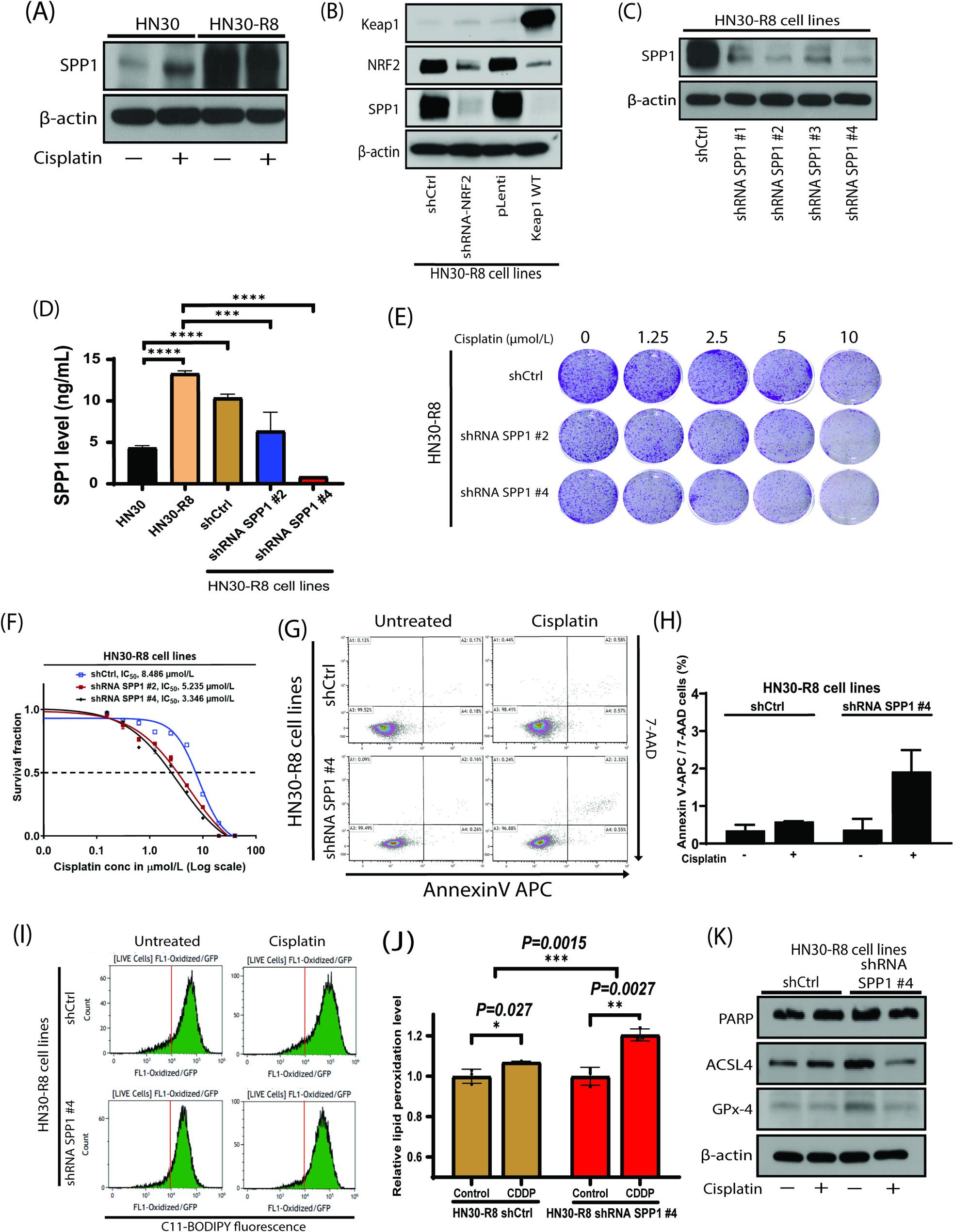
Downregulation of SPP1 enhances cisplatin sensitivity in vitro in cisplatin resistant HNSCC through induction of ferroptosis. **A**, Western blot demonstrates high expression of SPP1 during acquired CDDP resistance in HN30-R8 HNSCC cells compared to HN30 CDDP sensitive parental cells. **B**, Restoration of KEAP1 or knockdown of NRF2 modulates SPP1 expression in CDDP resistant HNSCC cells. **C**, Western blot verifies downregulation efficiency of SPP1 in stable CDDP resistant cells (HN30-R8). **D**, Bar graph depicting levels of SPP1 determined by ELISA in CDDP sensitive (HN30), resistant (HN30-R8) and their SPP1 knockdown derivatives. **E** and **F**, Representative images of clonogenic survival and curves demonstrating increased sensitivity to CDDP in HN30-R8 cells stably expressing shRNA SPP1 after treatment with the indicated doses of cisplatin. **G** and **H**, Annexin V-APC/7-AAD–positive staining (percentage of dead cells) confirming induction of low levels of apoptosis in SPP1 knockdown HN30-R8 cells treated with CDDP (8 µmol/L) for 48 hr. **I**, Fluorescence level of intracellular oxidized C11-BODIPY (581/591) measured by Flow cytometry. **J**, Bar graph showing increased lipid peroxidation levels calculated from the fluorescent integrated density of the oxidized BODIPY indicates ferroptosis in HN30-R8 cells expressing SPP1 shRNA following treatment with CDDP (8 mol/L) for 48 hr compared to untreated ShCtrl control cells. **K**, Western blot demonstrates decreased protein level of anti-ferroptotic markers, ACSL4 and GPX4 and absence of apoptotic marker, PARP cleavage. Data shown are representative of three independent experiments. Bar graphs are mean ± SEM, unpaired student t-test and two-way ANOVA respectively. **P < 0.001, ***P< 0.0001, *P=0.027, **P=0.0027, ***P=0.0015. Experiments were performed in triplicates and repeated 2 times

To investigate whether Nrf2-activated SPP1 contributes to cancer cell growth and cisplatin resistance in HNSCC, we initially evaluated the SPP1 expression levels in cisplatin sensitive (HN30) and resistant (HN30R-8) cell lines following treatment with cisplatin (CDDP) as indicated. Western blot analysis showed that acquired cisplatin resistance was associated with increased SPP1 protein levels in HN30-R8 cells compared to HN30 cells (Fig. 1A). Restoration of wt*KEAP1* or loss of NRF2 is accompanied by reduction in SPP1 protein levels in HN30-R8 cells, suggesting that SPP1 is one of the primary downstream targets modulated by dysregulated KEAP1/NRF2 signaling axis during acquired cisplatin resistance in HNSCC (Fig. 1B). We showed in a previous publication that re-expression of KEAP1 increased cisplatin sensitivity in vitro and in vivo in cisplatin resistant HN30-R8 model [10]. We next utilized SPP1 shRNA and control lentiviral vectors to generate stable SPP1-knockdown HN30-R8 and PCI13-wtp53R cell lines (Fig. 1C, Supplementary Fig. S1A and B) respectively. Western blot analysis demonstrated successful knockdown of SPP1 in these cell lines and HN30-R8 cells with SPP1 shRNA #2 and #4 were considered for further analysis (Fig. 1C). Because SPP1 is a secretory protein, we confirmed its level by ELISA in conditioned media prepared from these cells (Fig. 1D). Suppression of SPP1 enhanced cisplatin sensitivity and reduced the CDDP IC_50_ from 8.48 µmol/L in cells with lentiviral control (shCtrl) vector to 5.23 and 3.34 µmol/L in cells with lentiviral shRNA SPP1 #2 and #4 vectors respectively (Fig. 1E and F). Sensitivity to cisplatin was also observed in the PCI13-wtp53R cells expressing shRNA SPP1 #2 and #4 compared to the parental HN30 and shCtrl cells (Supplementary Fig. S1C and D). This result is consistent with published data demonstrated that SPP1 increased cisplatin resistance in lung cancer cells [29]. To determine how suppression of SPP1 sensitized cells to CDDP, mechanisms of cell death were assessed. SPP1 depletion has minimal effect on apoptosis (Fig. 1G and H). We next evaluated if suppression of SPP1 increased lipid peroxidation and induced ferroptosis in CDDP resistant HNSCC cells. Compared to control cells, cisplatin resistant HN30-R8 and PCI13 cells expressing shRNA SPP1 had a shift in excitation and emission upon oxidation (Fig. 1I) and significantly higher levels of lipid peroxidation upon CDDP exposure (Fig. 1J, Supplementary Fig. S1E and F), indicating cell death through ferroptosis. Treatment with the ferroptosis inhibitor ferrostatin-1 rescued SPP1-knockdown HN30-R8 cells from cell death (Supplementary Fig. S2A and B), confirming that ferroptosis is directly involved in this process. Furthermore, the lack of cleaved PARP1, a marker of apoptosis, and the decreased protein levels of the inhibitory ferroptosis markers, GPX4 and ACSL4 determined by western blot analysis confirmed induction of ferroptosis in the cells tested (Fig. 1K).

### Knock-down of SPP1 reduces in vitro colony growth and invasion in HNSCC cisplatin-resistant cells

**Fig. 2:**
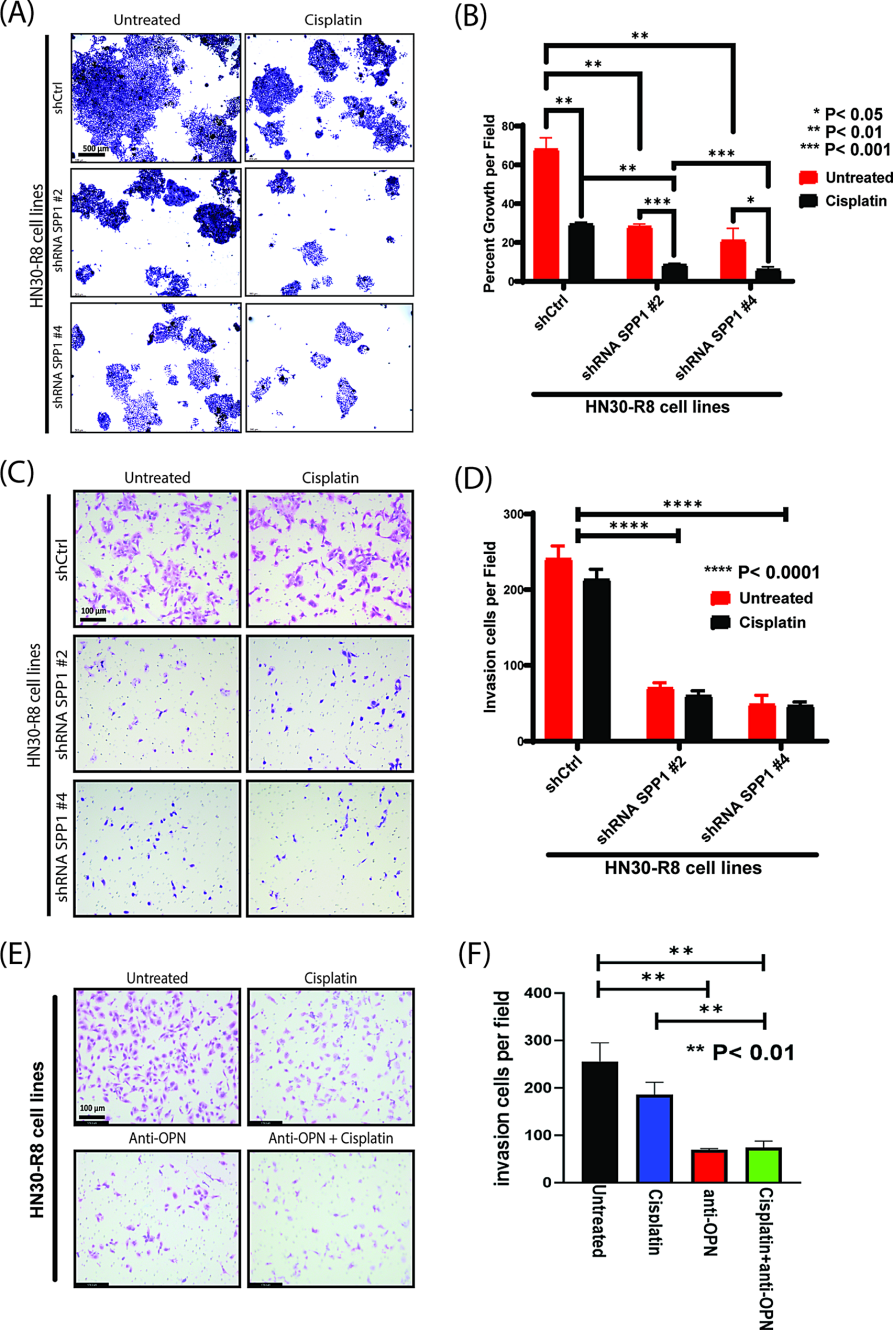
Inhibition of SPP1 reduces in vitro colony growth and inhibits cell invasion in HNSCC cisplatin-resistant cells. The HN30-R8 and their shRNA SPP1 derivatives were cultured in presence and absence of CDDP (8 mol/L) for 48 h and subjected to colony growth on soft agar and Matrigel invasion assays as described in Methods. **A** and **B**, Knock-down of SPP1 alone or with CDDP treatment decreases the colony growth on soft agar. **C** and **D**, The invasion ability of HN30-R8 cells expressing shRNA SPP1 is significantly decreased compared to the shCtrl control cells. **E** and **F**, Cell invasion is inhibited following neutralization of the SPP1 with the anti-SPP1(osteopontin) antibody and in the presence of CDDP in HN30-R8 CDDP resistant cells. Data shown are representative of three independent experiments (*N* = 3). Error bars are mean ± SEM, unpaired student t-test and two-way ANOVA respectively. *P < 0.05, *P < 0.01, ***P < 0.001, ****P < 0.0001. The scale bar is 100 μm. Images were taken using Leica DMLA microscope

Since SPP1 has been shown to promote tumor cell migration and invasion [12], colony formation and transwell migration assays were performed as previously described, to examine whether SPP1 downregulation exerted similar effect on CDDP-resistant HNSCC cancer cells. Compared to cells with lentiviral vector control (HN30-R8 shCtrl), the colony growth was significantly reduced in HN30-R8 shRNA SPP1 and this inhibitory effect is further enhanced upon treatment with CDDP (8 µmol/L) (Fig. 2A and B). Suppression of SPP1 inhibited cell invasion in both untreated and treated HN30-R8 and PCI13-wtp53-R cells (*P* < 0.0001) (Fig. 2C and D, Supplementary Fig. S1G and H). Similar results were obtained with anti-osteopontin antibody treatment of HN30-R8 cells (Fig. 2E and F).

### SPP1 targeted suppression potentiates sensitivity to cisplatin and inhibits tumor growth and metastasis in vivo in cisplatin-resistant oral cancer model

**Fig. 3:**
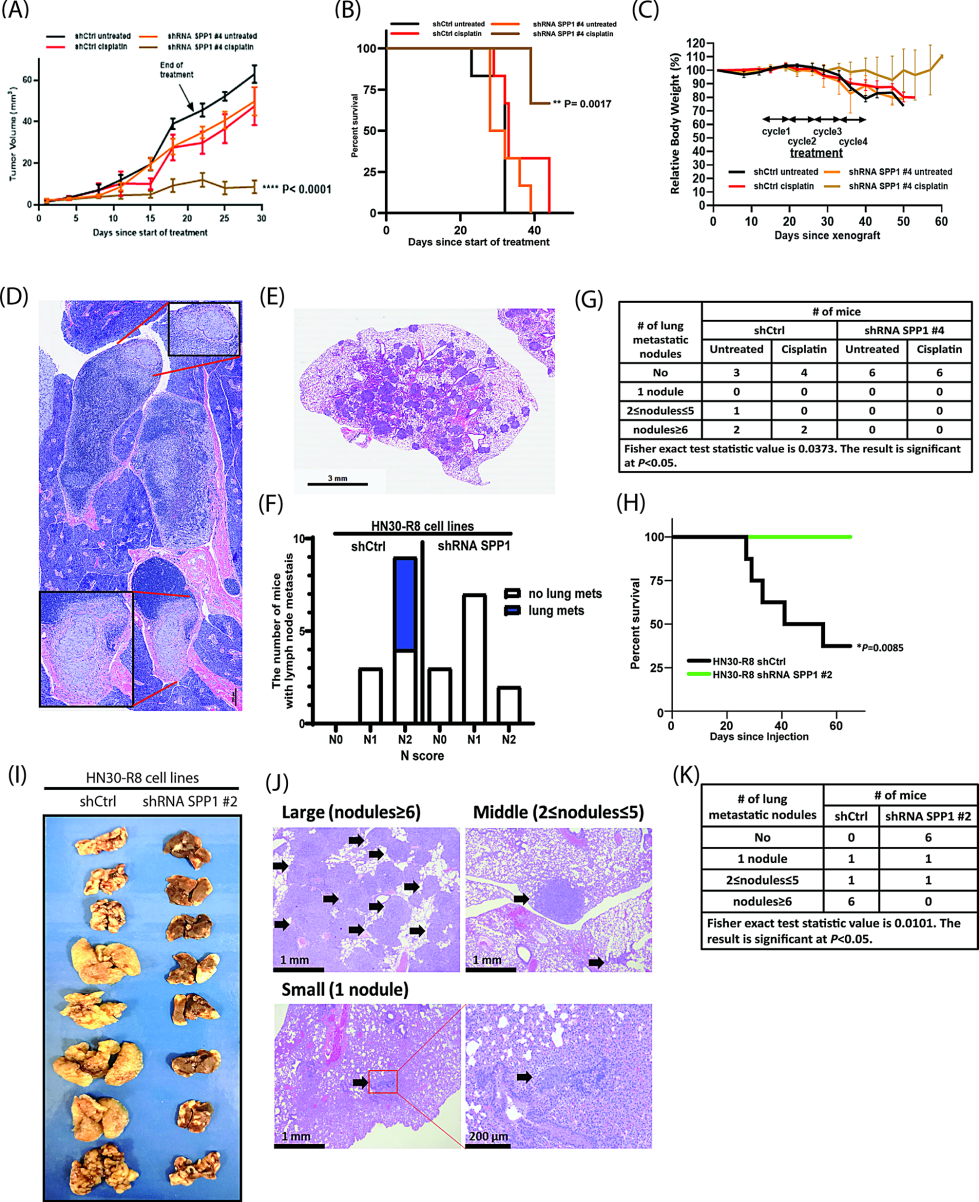
SPP1 targeted suppression enhances cisplatin sensitivity and reduces tumor growth and metastasis in vivo in cisplatin resistant oral cancer model. HN30-R8 cells stably expressing shRNA SPP1 #4 and shCtrl constructs were orthotopically injected into the tongues of male athymic nu/nu mice treated intravenously via tail injection with 4 mg/kg of CDDP for 4 weeks as indicated. Each treatment group contains 9–12 mice. Tumor growth was measured and reported as tumor volume means ± SEM. **A**, Tumor growth curves calculated after 4 weeks of injection and treatment. Statistical analyses were performed by a two-way ANOVA test. ****P < 0.0001 CDDP treated HN30-R8 shRNA SPP1 #4 tumor bearing mice versus all other treatment groups. **B** and **C**, Kaplan–Meier survival curve and percent of body weight loss of mice from **A**. The death of animals occurred when tumor compromised the animal welfare. **D** and **E**, Incidence of lymph node and lung tumor metastases in mice assessed microscopically by hematoxylin and eosin staining. **F**, Bar graph depicting the impact of SPP1 knockdown on the number of lymph node (N score) and lung tumor metastases present concurrently in mice. **G**, Table summarizes the number of mice with lung metastatic nodules, where No (indicates no nodule present). **H**, Kaplan–Meier survival curve of mice injected with HN30-R8 (shCtrl) and HN30-R8 (shRNA SPP1 #2 construct) cells through the tail vein. **I**, Macroscopic nodules can be seen in the representative images from the lungs of mice injected with the tumor cells via tail vein. **J** and **K**, respective microscopic images and Table depicting the number of the lung metastatic nodules, and their sizes appearing in mice following tail vein injection with the tumor cells. All in vivo data were expressed as ± standard error mean (± SEM) and Log-rank, *P < 0.05 was considered significant for mice survival. The Fisher exact test statistic value, *P < 0.05 was used to detect significant difference in lymph node and lung metastasis in mice

To evaluate the impact of altered SPP1 on CDDP sensitivity in vivo, we injected the HN30-R8 stably expressing shRNA SPP1 #4 and shCtrl vectors into the tongues of athymic nu/nu mice followed by CDDP treatment as indicated. As expected, no significant response was observed upon CDDP treated bearing HN30-R8 shRNA Ctrl transfected cells (Fig. 3A). Consistent with published data [30], tumor growth in untreated mice orally injected with cells expressing shRNA SPP1 #4 vector was slightly decreased compared to the untreated shRNA Ctrl control group (Fig. 3A). However, significant tumor growth inhibition was observed following CDDP treatment in mice injected with shRNA SPP1 #4 cells, compared with all other treated or untreated groups (*P* < 0.0001; Fig. 3A). CDDP treatment was associated with improved animal survival in mice bearing shRNA SPP1 #4 cells as compared to the untreated group and animals in the CDDP treated and untreated HN30-R8 shRNA controls (*P* = 0.0017; Fig. 3B). Body weight was not significantly different between the groups with or without CDDP treatment (Fig. 3C). We assessed the effect of SPP1 down-regulation on the development of lung metastases of CDDP resistant HNSCC cells in vivo. Mice were injected orally with the shCtrl and ShRNA SPP1 #4 cells followed by treatment with CDDP as indicated. A total of 12 mice (6 untreated and 6 CDDP treated) were used in each group. Histological H&E staining was utilized to evaluate for the presence of microscopic metastases in the lymph node and lung sections obtained from these mice. We defined N0 as non-metastatic, N1 as simple metastasis (metastasis to one lymph node, and N2 as multiple metastases (metastases to more than one lymph) in cervical lymph node (Fig. 3D-F). Lung metastatic nodule counts in mice are tabulated and shown in Fig. 3G. Mice harboring HN30-R8 shCtrl tumors had higher rates of cervical lymph node metastases (9 mice with N2 score), and 5 of the 9 mice with staged as N2 in this group had multiple lung metastases (Fig. 3D-F and Supplementary Fig. S4A). Mice harboring tumors with SPP1 knockdown had fewer number N2 (2/12 mice) metastases (Fig. 3D-F and Supplementary Fig. S4A). None of the mice with SPP1 knockdown were found to have lung metastasis (Fig. 3F-G). Additionally, the accelerated rate of distant metastasis was further confirmed in the tail vein metastatic model using tumor cells expressing the shCtrl vector (8 mice) and other shRNA SPP1 construct #2 (8 mice) (Fig. 3H). While all mice in the control group (shCtrl) had lung metastases, only two mice in the shRNA SPP1 #2 group were found to have a very low number metastatic nodules in their lungs (Fig. 3I and K, and Supplementary Fig. S4B). A significant improvement in mouse survival (*p* = 0.0085) was observed in the SPP1 knock down group as compared to the HN30-R8 shRNA. SPP1 is known to modulate the tumor microenvironment through induction of oxidative stress [31]. Accordingly, we assessed the expression of oxidative stress and ferroptosis-associated markers, including GPX4 and ACSL4, in mice tongue tumors by immunohistochemistry. In response to cisplatin treatment, tumors with SPP1 knockdown exhibited a significant reduction in GPX4 expression and a concomitant increase in ACSL4 immunostaining compared with control tumors, consistent with our in vitro findings (Supplementary Fig. S3A and B). Taken together, these data suggest that dysregulated SPP1 plays a critical role in tumor progression and metastasis in NRF2 activated cisplatin resistant HNSCC.

### Pharmacological inhibition of SPP1 enhances cisplatin sensitivity in vivo in CDDP resistant oral cancer model

**Fig. 4:**
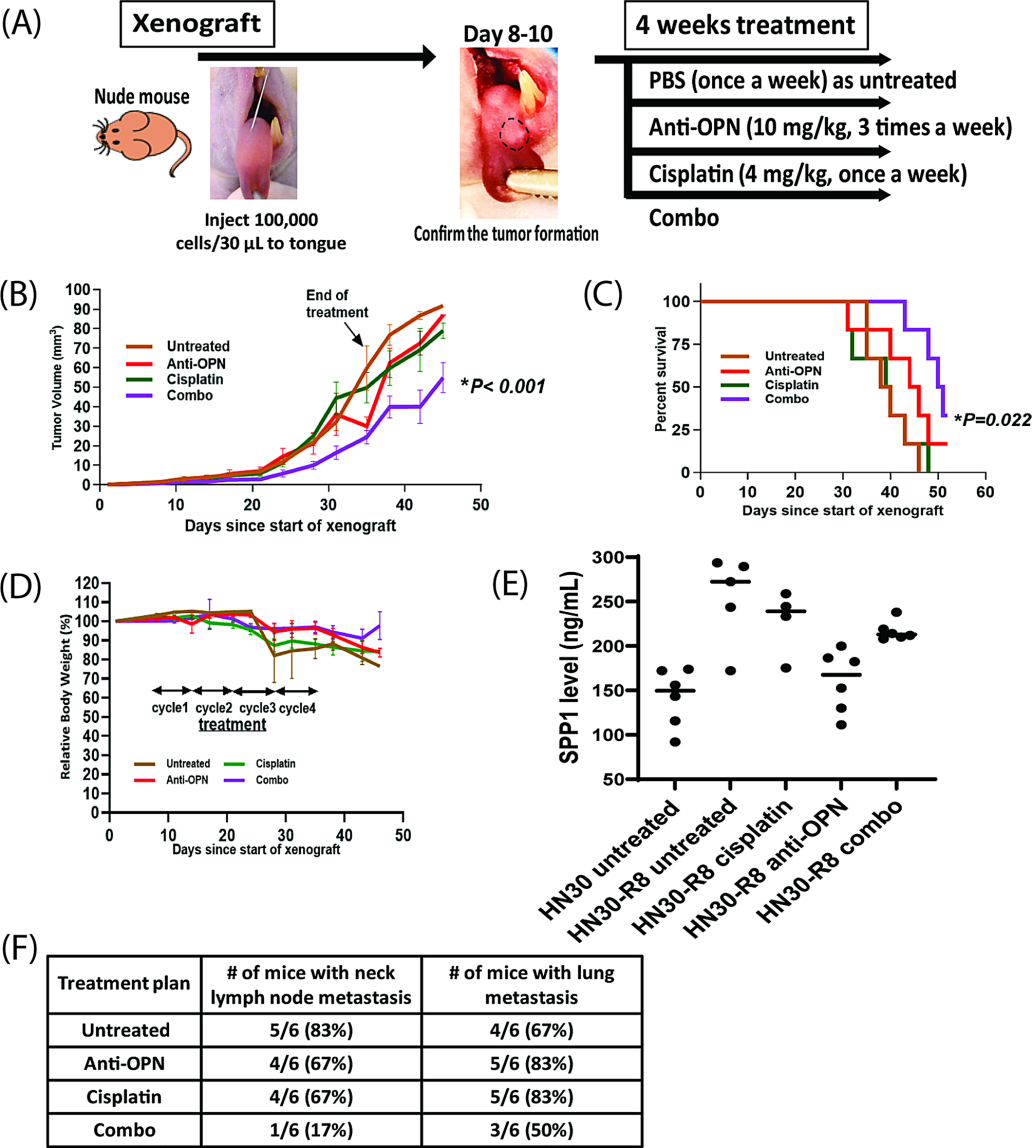
Pharmacological inhibition of SPP1 improves cisplatin sensitivity in vivo in cisplatin resistant oral cancer model. **A**, Schematic procedure of the in vivo mouse xenograft experiment and treatment schedule. Mice were injected with CDDP resistant HN30-R8 cells and after tumor formed, mice were treated with CDDP, anti-OPN, and in combination as indicated. **B**, Tumor growth curves calculated after 4 weeks of injection and treatment with drugs. **C**, Kaplan–Meier survival curve demonstrating improved survival of mice following combination of anti-OPN and CDDP. **D**, Percent body weight loss among the treatment groups. **E**, SPP1 levels determined by ELISA-based assay in plasma prepared from mice blood samples collected following treatment with drugs. The plasma samples were diluted 100 times in the ELISA dilution buffer. **F**, Table showing microscopic evaluation of incidence and percentage of tumor metastatic nodules in mice lymph nodes and lungs sections stained with H&E as indicated. A total of mice (*N* = 6) in each treatment group was used. All in vivo data were expressed as ± SEM and two-way ANOVA and Log-rank tests, *P < 0.05 were considered significant for tumor growth reduction and mice survival, respectively

As SPP1 suppression led to significant tumor growth reduction and improved sensitivity to cisplatin in vivo, we sought to pharmacologically target SPP1 and evaluate its impact on tumor growth and metastasis using a commercially available anti-osteopontin (SPP1) antibody. HN30-R8 tumors were randomly assigned to one of 4 treatment groups: (1) control, (2) CDDP, (3) anti-OPN antibody and (4) anti-OPN antibody in combination as indicated in the protocol outlined in Fig. 4A. Antibody dose (10 mg/kg) and frequency of administration was chosen based on the in vitro data (Fig. 2E). No significant anti-tumor efficacy was observed with the anti-OPN treatment alone at this selected dose compared to either untreated or CDDP treated groups. However, the combination of CDDP and anti-OPN antibody was associated with delayed tumor growth and improved animal survival (Fig. 4B and C). No significant body weight loss was seen among the treatment groups, indicating that the combination was well tolerated (Fig. 4D). Plasma levels of OPN (SPP1) were significantly reduced after treatment with anti-OPN antibody given alone or in combination with CDDP compared to untreated mice or those treated with CDDP alone (Fig. 4E). OPN (SPP1) levels were decreased in the anti-OPN and combination treatment groups to a level comparable to that obtained from mice implanted with the CDDP sensitive parental HN30 cell line (Fig. 4E). These data confirmed that the anti-OPN dose used was able to deplete SPP1 and prevent binding to its cognate receptors in vivo. Additionally, the effect of anti-OPN antibody and its combination with CDDP on tumor progression and metastasis was microscopically evaluated in the mice lymph nodes and lungs sections stained with H&E as indicated. Consistent with published report [32], only the combination of anti-OPN antibody and CDDP was able to reduce the number of mice with cervical lymph nodes (1 out of 6 mice; 17%) and lung metastasis (3 out of 6 mice; 50%) compared to other treatment groups (Table in Fig. 4F and Supplementary Fig. S3A and B), suggesting that OPN (SPP1) plays an important role in tumor growth and regional and distant metastasis.

### SPP1 gene expression is a poor prognostic factor that correlates with *NRF2/KEAP1* mutational status and aggressive clinical features in HNSCC

**Fig. 5:**
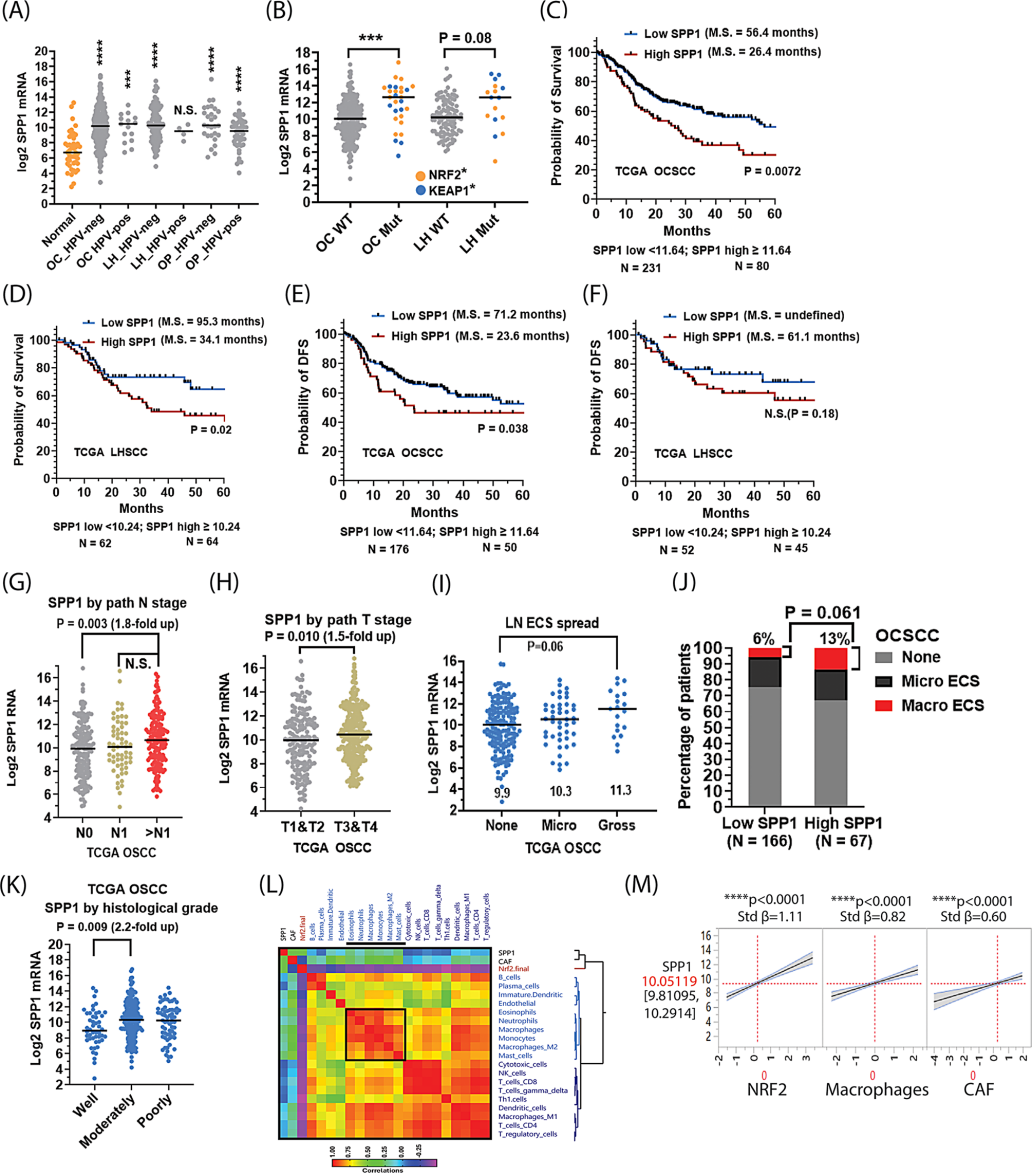
SPP1 correlates with poor prognosis, *NRF2/KEAP1* mutational status, and aggressive clinical features in HNSCC. **A**, Levels of SPP1 mRNA are significantly higher in oral cavity (OC) squamous cell carcinoma (OCSCC) and laryngeal / hypopharyngeal (LH) squamous cell carcinoma tumors compared to normal samples from the TCGA cohort, regardless of HPV association. **B**, SPP1 expression is elevated in tumors harboring mutations in either NRF2 (orange symbols) or KEAP1 (blue symbols) genes. **C**, OCSCC tumors with elevated SPP1 mRNA expression (cohort cutoff determined by recursive portioning) have significantly lower median overall survival time. **D**, LHSCC tumors with elevated SPP1 mRNA expression (cohort cutoff determined by recursive portioning) have significantly lower median overall survival time. **E**, OCSCC tumors with elevated SPP1 mRNA expression have significantly lower median disease-free survival (DFS) times. **F**, LHSCC tumors with elevated SPP1 mRNA expression have significantly lower median DFS times. **G**, OSCC patients with pathological lymph node stage > N1 have significantly higher SPP1 mRNA expression compared to node negative (N0) patients. **H**, OCSCC patients with more advanced T stages (T3 & T4) have significantly elevated SPP1 mRNA expression. **I**, Primary tumors with higher levels of SPP1 mRNA had a greater incidence of gross extracapsular lymph node extension. **J**, Primary OCSCC tumors associated with microscopic (micro) or gross extracapsular lymph node spread had a trend towards elevated SPP1 mRNA expression. **K**, Elevated expression of SPP1 mRNA was associated with less differentiated OCSCC tumors. *P ≤ 0.05, **P ≤ 0.01, ***P ≤ 0.001, ****P ≤ 0.001. **L**, Hierarchical clustering of the cross-correlation coefficients featuring modules highly co-correlated among a subset of leukocytes including eosinophils, neutrophils, monocytes, mast cells, and macrophages which all moderately correlated with SPP1 expression levels. **M**, Regression model revealing that NRF2 is the strongest predictor of SPP1 (****p < 0.00001), followed by macrophages. (****p < 0.00001), and CAF (****p < 0.00001)

We analyzed RNAseq data from the TCGA HNSCC cohort to investigate how SPP1 gene expression correlates with clinical features and prognosis. HNSCC tumors were initially stratified by subsite, including oral cavity SCC (OSCC), laryngeal/hypopharyngeal SCC (LHSCC), and oropharyngeal SCC (OPSCC) as well as by HPV status, to compare SPP1 expression with adjacent normal samples. Compared to normal samples, levels of SPP1 expression were significantly increased in tumors from nearly all the subsites, regardless of HPV status, with the exception of HPV-positive LHSCC (Fig. 5A). Next, we examined whether mutations in either *NFE2L2* or *KEAP1* were associated with increased SPP1 expression because SPP1 is a known downstream target of NRF2 activation. Collectively, tumors with a mutation in either one of these genes had significantly elevated average SPP1 mRNA (*P* < 0.001, Fig. 5B) in the OCSCC cohort, and a similar trend was found among LHSCC tumors that nearly reached significance. OCSCC and LHSCC patients with high SPP1 expression in their tumors, determined through recursive partition analysis, had significantly reduced overall median survival times (Fig. 5C and D), which were also associated with reduced median disease-free survival times (Fig. 5E and F). Consistent with these findings, OCSCC tumors from patients with a higher N stage (> N1) and T stage (T3 &T4) had significantly increased average expression of SPP1 (Fig. 5G and H), and reciprocally patients with tumors categorized as high SPP1 expression were associated with an increased frequency of gross (e.g., Macro) extracapsular spread (Fig. 5I). Likewise, tumors associated with the presence of gross extracapsular spread had a tendency towards increased SPP1 expression (Fig. 5J). The average SPP1 expression of poorly and moderately differentiated tumors was increased relative to well differentiated tumors, with the latter more than two-fold higher (*P* = 0.009, Fig. 5K). Because SPP1 can be expressed by macrophages, cancer-associated fibroblasts (CAF), tumor cells, and possibly other cells present in the tumor microenvironment we examined the putative relationships between SPP1 expression, the presence of various cell types, and survival. To untangle the possible connections between SPP1 expression and cell types in the tumor microenvironment we first performed single sample gene set enrichment analysis (ssGSEA) on TCGA OCSCC samples using published gene lists specific for enrichment of 19 different cell types that included various leukocyte subsets, endothelial cells, CAF, and cross correlated the scores with SPP1 mRNA expression and also NRF2 activation scores (Supplementary Table S1). Hierarchical clustering of the cross-correlation coefficients enabled visualization of feature modules that behaved similarly (Fig. 5L), where we observed high co-correlation among a subset of leukocytes including eosinophils, neutrophils, monocytes, mast cells, and macrophages―all moderately correlated with SPP1 levels. Among these we chose macrophages to represent this leukocyte group in building a multiple linear regression model of SPP1 expression because of prior literature documenting SPP1 expression in macrophages. We also examined NRF2 scores and CAF scores as additional predictors because they appeared to independently correlate with SPP1 from the heatmap. The regression model (Fig. 5M) revealed that NRF2 was the strongest predictor of SPP1 (β = 1.11, ****p < 0.0001), followed by macrophages (β = 0.82, ****p < 0.0001), and CAF (β = 0.60, ****p < 0.0001). The β values are the standardized regression coefficients in the model to demonstrate relative contributions and the multivariate factors have significant effects as indicated by the asterisks above the regression coefficients. We performed univariate and multivariate cox regression considering SPP1, macrophage score, NRF2 score, and CAF score as predictors of overall survival using the TCGA OCSCC cohort (Supplementary Table S2). SPP1 was the only variable to correlate with overall survival in either the univariate (β = 0.237, HR = 1.27, *P* = 0.0078) or multivariate (β = 0.277, HR = 1.32, *P* = 0.0078), indicating that high SPP1 expression was an independent significant predictor of poor overall survival. The overall and disease-free survival were also assessed in HPV-positive OPSCC patients stratified by SPP1 expression. In contrast to HPV-negative tumors, elevated SPP1 mRNA levels in HPV-positive tumors were associated with a significantly longer median overall survival (Supplementary Fig. S6A-D). Regression analysis identified macrophages as the strongest predictors of SPP1 expression, whereas NRF2 signature and cancer-associated fibroblasts (CAFs) exhibited relatively weak associations (Supplementary Fig. S6E).

### Reverse phase protein array (RPPA) analysis identifies protein analytes and metastatic signaling pathways downstream of SPP1 expression

**Fig. 6:**
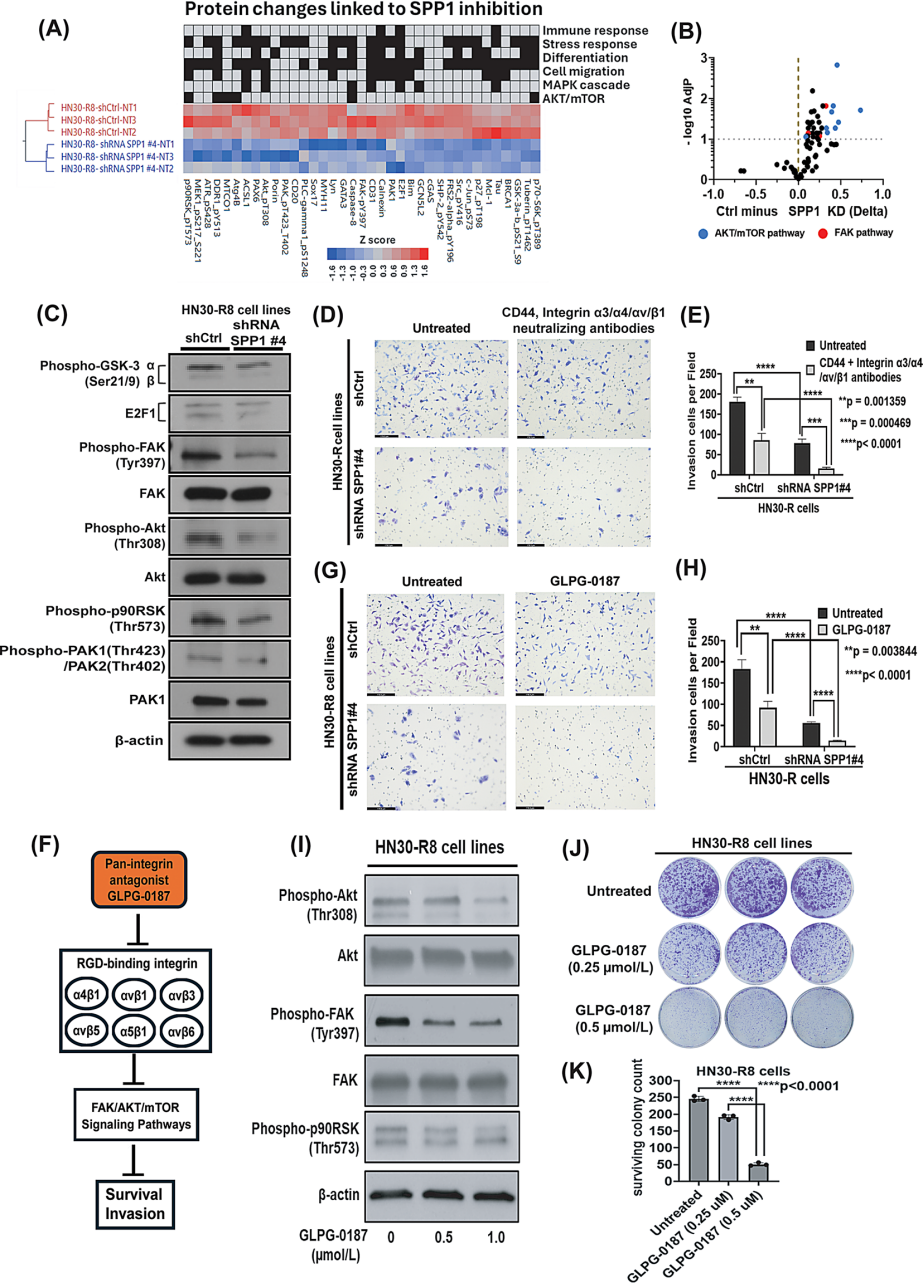
Reverse phase protein array (RPPA) analysis identifies protein analytes and metastatic signaling pathways downstream of SPP1 expression. **A**, Heatmap of select analytes measured by RPPA after one-way unsupervised hierarchical clustering, showing that samples clustered by the status of SPP1 KD. Gene Ontology (GO) enrichment was performed on all analytes found significantly different after SPP1 KD and analytes from select GO pathways found to be significantly enriched were included in the cluster analysis and are annotated by GO process with black squares above the heatmap. **B**, Volcano plot showing negative log10 adjusted P values (y-axis) verses differences in RPPA values (x-axis) measured for analytes derived from HN30R8 cells after infection with either control shRNA (shCtrl) or shRNA SPP1 knockdown (SPP1 KD. Positive delta values indicate analytes that decrease after SPP1 KD compared to control. Analytes in the AKT / mTOR pathway are represented with blue symbols, while analytes from the FAK pathway are represented with red symbols. A doted horizontal line at Y = 1 corresponds to a minimum false discovery rate of 0.1. **C**, Western blot analyses confirming differential expression of most of these proteins, including AKT/ mTOR and FAK among the control and SPP1 KD cell lines. **D** and **E**, Invasion assays showing that the invasive capacity of HN30-R8 cisplatin-resistant cells expressing shCtrl or shRNA SPP1 is significantly reduced following neutralization of CD44 and integrin receptors using a combination of anti-CD44 and integrin α3/α4/αv/β1 antibodies. **F**, Schematic representation illustrating the binding of the pan-integrin antagonist GLPG-0187 to the RGD motif of integrins and its inhibitory effects on FAK, AKT, and mTOR signaling. **G** and **H**, Invasion assays demonstrating a decreased number of invasive HN30-R8 cells expressing shCtrl or shRNA SPP1 following treatment with the pan-integrin antagonist GLPG-0187. **I**, Western blot analyses showing reduced phosphorylation of FAK, AKT, and p90RSK in HN30-R8 cisplatin-resistant cells following GLPG-0187 treatment. **J–K**, Representative clonogenic survival images and corresponding survival curves demonstrating increased sensitivity of HN30-R8 cells to the indicated doses of GLPG-0187. Data are representative of three independent experiments (*N* = 3), each performed in duplicate or triplicate as appropriate. Error bars represent mean ± SEM. Statistical analyses were performed using an unpaired Student’s t-test or two-way ANOVA, as appropriate. Scale bar, 179.3 μm. Images were acquired using a Leica DMLA microscope

Binding of SPP1 to its receptors triggers a cascade of intracellular signaling pathways that regulate cell proliferation and tumor progression. Therefore, to investigate the molecular mechanisms underpinning SPP1 mediated tumor progression and cisplatin resistance, we employed reverse phase protein array (RPPA) analysis to identify differences in total proteins and phosphoproteins after SPP1 knockdown in HN30-R8 cells. Compared to the shRNA control, SPP1 knockdown caused significant decreases (FDR < 0.1) in 37 out of 72 analytes assayed, which included multiple proteins and phosphoproteins from the AKT/mTOR pathway (pAKT_T308, p70-S6K_T389, p90RSK_T573, pTSC2_T1462, pGSK3α-β_S21/9), the MAPK pathway (pPAK_T403/T423, PAK1, pMEK1_S217/S221), and diminished FAK activation (Supplementary Table S3 and Fig. 5A and B). Gene ontology enrichment analysis revealed that the analytes downregulated by inhibition of SPP1 were enriched for cellular and tumor promoting processes involved in cell migration, communication, differentiation, stress response, and immune response activation (Supplementary Table S3 and Fig. 6B). Reduction in activation of AKT, p90RSK, PAK1 and FAK were confirmed by western blotting as well as loss of GSK3 on an inhibitory site (S9/S21) ordinarily maintained by AKT (Fig. 6C). Increased activity of these proteins is associated with tumor cell survival and metastasis in several cancer types, making them potential protein targets in HNSCC. These data suggest that SPP1 inhibition causes significant decrease in relevant oncogenic and metastatic signaling pathways and functions during cisplatin resistant in HNSCC. We directly interrogated SPP1 receptor function using blocking antibodies and the pan-integrin antagonist GLPG-0187. Neutralization of CD44 and integrins (α3/α4/αv/β1) significantly reduced invasion of cisplatin-resistant HN30-R8 cells expressing either shCtrl or shSPP1 (****p < 0.0001; Fig. 6D and E). GLPG-0187, which targets the RGD-binding motif of integrins (Fig. 6F), similarly and robustly suppressed invasion in both cell populations (****p < 0.0001; Fig. 6G and H). GLPG-0187 treatment markedly reduced phosphorylation of FAK, AKT, and p90RSK and increased sensitivity of cisplatin-resistant HN30-R8 cells to integrin inhibition (Fig. 6J and K). Collectively, these findings indicate that SPP1-mediated phenotypes require signaling through the integrin/CD44 axis.

### Visium spatial transcriptomic analysis in metastatic HNSCC tumor reveals a potential mechanistic interplay between tumor-derived SPP1, integrins and CD44 receptors

**Fig. 7:**
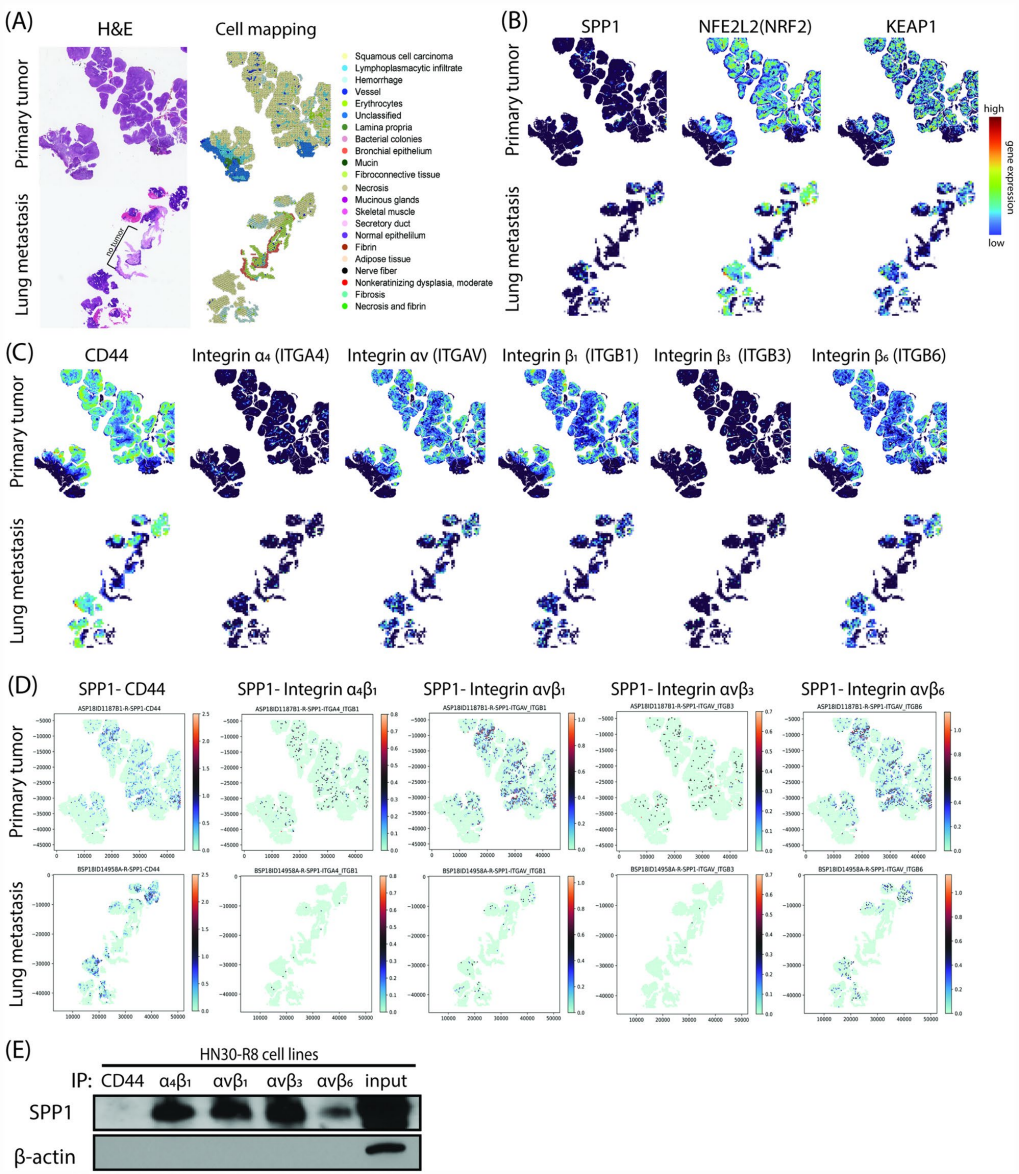
Visium spatial transcriptomic analysis in metastatic HNSCC reveals a potential mechanistic interplay between tumor-derived SPP1, integrins and CD44 receptors. **A**, Hematoxylin and eosin staining and UMAP plots showing the histological annotation of the H&E -stained Visium slides and cell types in patient primary and metastatic lung tumors. **B**, Near super-pixel resolution analysis using iSTAR and visualization of differential expression of SPP1, NFE2L2 (NRF2), KEAP1 and KEAP1 in primary and metastatic lung tumors. **C**, Near super-pixel resolution analysis using iSTAR and visualization of the differential expression of SPP1 receptors in primary and metastatic lung tumors. **D**, COMMOT clustering analysis and similar communication intensity signals demonstrating possible interaction between SPP1 and its receptors, CD44 and integrins in primary and metastatic lung tumors in HNSCC patients. **E**, Western blot analysis showing positive interaction of SPP1 with CD44 and several integrin receptors in vitro in CDDP-resistant HN30-R8 cell lines. **F**, Proposed signaling model summarizing the oncogenic role of SPP1 in HNSCC. We hypothesized that under NRF2-stressed conditions, hyperactivated SPP1 interacts with specific class of α/β integrins and CD44 in the tumors and activate downstream signaling effectors including AKT/mTOR, MAPK and FAK proteins. Consequently, this interaction between SPP1 and its receptors in cisplatin-resistant HNSCC enhances oncogenic cellular responses and inhibits ferroptosis, ultimately contributing to therapy resistance, tumor progression, and metastasis

The continuous interplay between tumor cells and the tumor microenvironment (TME) strongly affects tumor development, disease progression, metastasis, and responses to therapeutic interventions. The spatial composition and microenvironmental ecological niche within tumors have been reported to be associated with TME remodeling and tumor metastasis [33]. In this study we have shown that elevated level of SPP1 is associated with the development of cisplatin resistance, tumor progression and metastasis in vivo in NRF2-hyperactivated HNSCC. This is significant because recent studies have highlighted the pivotal role of SPP1 in the progression of malignant tumors through modulation of TME [34, 35]. Therefore, we sought to investigate whether the metastatic potential of cisplatin-resistant HNSCC is linked to a spatial interaction between NRF2-activated SPP1 signaling and the TME. We performed Visium spatial transcriptome (ST) on archival primary and metastatic FFPE tissue sample from one recurrent/metastatic head neck cancer patient. H&E staining and UMAP revealed distinct, color-coded cell types and pathologically annotated clusters (spots) from the ST dataset of primary and metastatic lung tumors. (Fig. 7A). As expected, the squamous cell carcinoma represented the majority of the cell type in these tumors (Fig. 7A). The top 3,000 highly variable genes (HVGs) were selected for Principal Component Analysis (PCA) and downstream unsupervised spot clustering. The iSTAR analysis in tumor samples identified a KEAP1/NRF2 gene signature in 400 out of over 1000 genes (Supplementary Table S4). Although, KEAP1 and NRF2 expression was similar in primary and metastatic tumors, the SPP1 expression was significantly higher in lung metastatic tumors compared to the primary tumor (Fig. 7B). This increased SPP1 expression is consistent with its known functional role in promoting tumor metastasis. SPP1 acts through binding to various cell surface integrins and CD44 receptors and activates downstream signaling pathways to promote tumor growth, invasion, and chemoresistance [16–18, 29, 36]. However, its interaction with these receptors during cisplatin resistance or tumor progression in HNSCC is not fully elucidated. COMMOT is a computational method which incorporates the spatial information and ligand-receptor interactions in spatial transcriptomic data. Here, we combined the iSTAR with the COMMOT to identify spatial cell-cell communication patterns in the HNSCC patient tumors from the gene expression, focusing on SPP1 and its cognate receptors. High expression levels of major SPP1 receptors including CD44 and several integrin subunits (α4, αv, β1, β3, and β6) were observed in the cell types in the patient primary and metastatic lung tumors (Fig. 7C), revealing a strong interaction between SPP1 and several integrins (α4β1, αvβ1, αvβ3, and αvβ6) in both primary and metastatic tumors (Fig. 7D). In HNSCC lung metastases, SPP1 appears to bind specifically to CD44, integrin αvβ1 and integrin αvβ6 (Fig. 7D). Immunoprecipitation analysis on CDDP-resistant HN30-R8 cell lines in vitro partly confirmed this interaction, showing strong co-immunoprecipitation of SPP1 with integrins α4β1, αvβ1, and αvβ3, but not with CD44, in CDDP-resistant HN30-R8 cells (Fig. 7E). Using immunohistochemical and immunofluorescence analyses, robust co-localization of SPP1 with integrin β1 and CD44 was observed in both primary tumors and lung metastases following treatment with cisplatin (Supplementary Fig. S7A–C and S8A–B), supporting a mechanistic interaction between tumor-derived SPP1 and integrin/CD44 receptors, as predicted by spatial transcriptomic and COMMOT analyses. This validation provides clear evidence for the interaction between SPP1 and its predicted integrin receptors and is consistent with recent publications [16–18]. Collectively, our research findings suggest that SPP1 interacts with integrins and CD44 in HNSCC tumors, potentially enhancing oncogenic signaling and contributing to therapy resistance, tumor progression and metastasis (Fig. 7F).

### Spatial annotation and gene set enrichment analyses identify high expression of SPP1 and distinct cancer HALLMARK pathways between primary and metastatic lung tumors in HNSCC patients

**Fig. 8:**
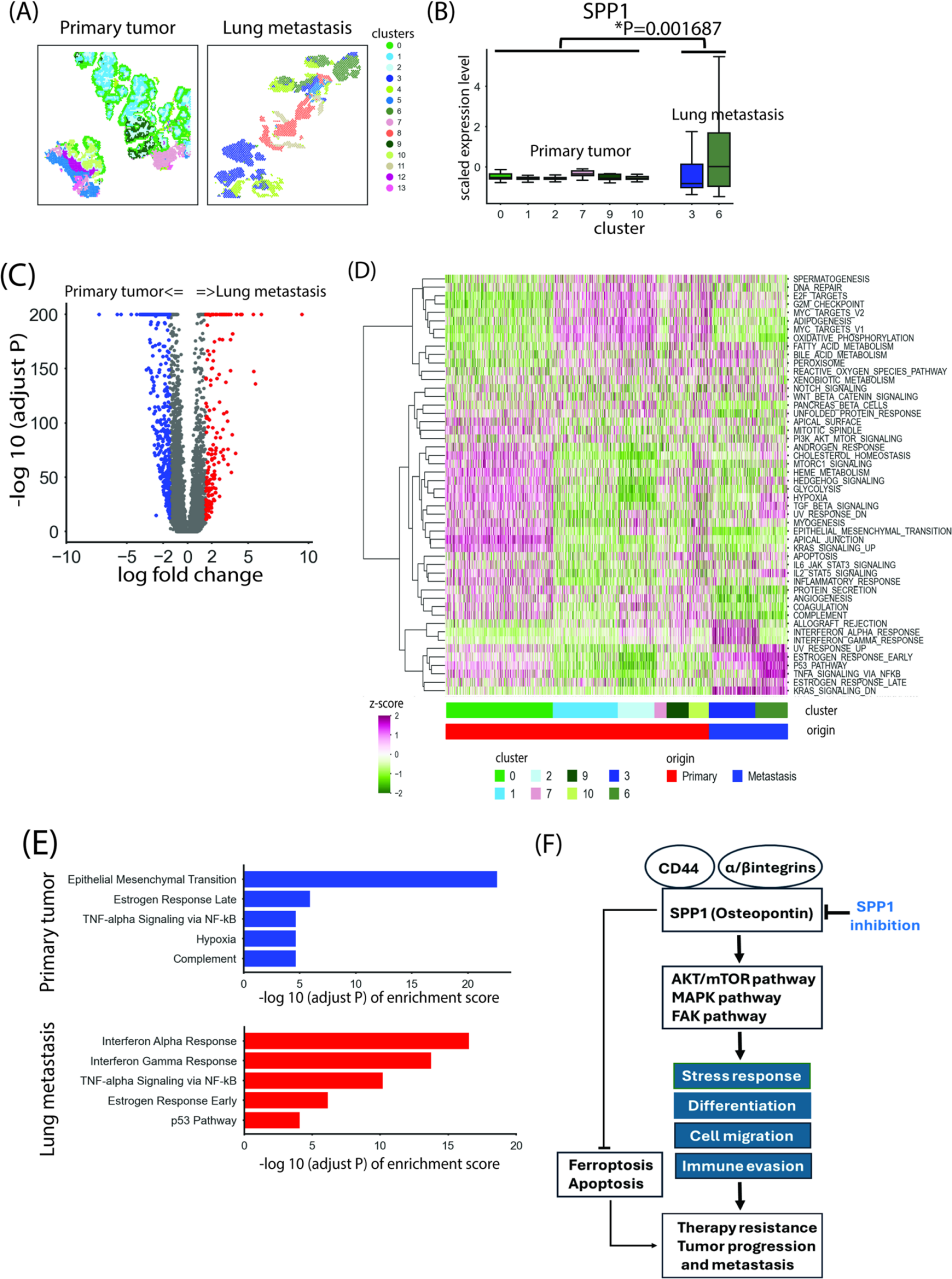
Spatial annotation and gene set enrichment analyses identify high expression of SPP1 and distinct cancer HALLMARK pathways between primary and metastatic lung tumors in HNSCC patients. **A**, Spots filtered by pathology annotation from the Visium slides analyzed by Seurat showing 14 cell clusters (0-13) in primary and metastatic lung tumors. **B**, Box plot showing scaled expression levels of SPP1 in each cell cluster (*p=0.001687, t-test). **C**, Volcano plot depicting the log fold change (logFC>1.5 and FDR <0.01) of the differentially expressed genes in primary and metastatic lung tumors. **D**, Heatmap showing HALLMARK enrichment analyses for 50 MSigDB ALLMARK gene sets profile obtained from the differentially expressed gene list of each cluster using the AUCell package. Red horizontal bar represents primary tumor with clusters (0, 1, 2, 7, 9, and 10) and blue horizontal bar represents metastatic lung tumor with clusters (3 and 6). **E**, Bar graph showing top five enriched gene signatures selected from the 50 MSigDB HALLMARK gene sets in primary and metastatic lung tumors. **F**, Proposed signaling model illustrating the oncogenic role of SPP1 in HNSCC. Under NRF2 stress, hyperactivated SPP1 binds α/β integrins and CD44 in tumors, activating AKT/mTOR, MAPK, and FAK signaling. This promotes oncogenic responses, suppresses ferroptosis, and drives cisplatin resistance, tumor progression, and metastasis in HNSCC

To analyze gene expression differences between paired primary and metastatic lung tumors in HNSCC patients, differentially expressed genes were identified within 14 cell clusters (cluster 0–13) using Seurat (Fig. 8A). The pathology annotation of the cell types and clusters in the primary and metastatic lung tumors was analyzed by Seurat (Supplementary Table S5). These genes were then annotated to understand their function and roles within each cluster. This approach allows for a detailed comparison of molecular changes occurring during tumor metastasis. The top 6 significantly expressed genes in each cluster were presented as bubble and annotation clustering plots (Supplementary Fig. S9). Our data further showed that clusters 0, 1, 2, 7, 9, and 10 were more prevalent in the primary tumor, while clusters 3 and 6 were the predominant features of the metastatic lung tumor (Supplementary Table S5). Notably, the SPP1 expression was higher in clusters 3 and 6 in the metastatic lung tumor (Fig. 8B). Only 525 genes with logFC > 1.5 and FDR < 0.01 were considered for further analysis. Therefore, 186 differentially expressed genes were significantly upregulated in the primary tumor and 339 genes in the metastatic lung tumor as shown in the volcano plot (Fig. 8C and supplementary Table S6). On the basis of these findings, we next performed functional HALLMARK and enrichment analyses of these target genes using the AUCell package to identify different gene signatures active in cells from primary and metastatic lung tumors (Fig. 8D). Primary tumor clusters showed significant enrichment of genes related to epithelial-mesenchymal transition (EMT) signatures, while metastatic lung tumor clusters exhibited enrichment of interferon response signatures (Fig. 8E and supplementary Table S7A and S7B). A proposed signaling model illustrating the potential oncogenic role of SPP1 in cisplatin resistance and tumor progression in HNSCC is presented in Fig. 8F.

## Discussion

Cisplatin-based concurrent chemoradiotherapy remains the first-line treatment for most patients with advanced head and neck squamous cell carcinoma (HNSCC) [7, 9]. However, cisplatin resistance contributes significantly to treatment failure, locoregional and distant metastasis, and cancer-specific mortality [7, 9]. Therefore, identifying predictive biomarkers of cisplatin response is crucial to reducing recurrence rates and mortality in HNSCC. In a previous study, we demonstrated that *SPP1*, a target gene of NRF2, is significantly upregulated both in vitro and in vivo in cisplatin-resistant HNSCC tumor models [9]. Several reports have also indicated that *SPP1* plays a key role in promoting invasion and metastasis in various solid tumors [12, 32, 37, 38]. In this study, using human cisplatin-resistant tumor cell lines stably expressing *SPP1* shRNA and patient-derived samples, combined with high-throughput proteomic and transcriptomic analyses, we systematically investigated the role of NRF2-induced *SPP1* in HNSCC progression.

Our findings clearly show that targeted suppression of *SPP1* restores sensitivity to cisplatin in resistant HNSCC cells, both in vitro and in vivo. This increased sensitivity appears to be driven by the activation of ferroptosis, a form of programmed cell death characterized by lipid accumulation and peroxidation through iron utilization [39]. These results suggest that NRF2-induced *SPP1* suppresses ferroptosis, thereby reducing cisplatin efficacy in resistant HNSCC. Moreover, we observed that silencing *SPP1* markedly impairs the ability of cisplatin-resistant HNSCC cells to invade and metastasize to lymph nodes and lungs in vivo. Consistent with this, we found that OCSCC tumors from TCGA cohort had an increased propensity for extra capsular spread in regional lymph nodes. Collectively, our preclinical data and analyses demonstrate that high SPP1 expression is a key mediator of disease progression in tumors, including those with NRF2 hyperactivation driven by a *KEAP1* mutation, leading to increased regional and distant metastasis. Importantly, regression analysis on the TCGA OCSCC data indicated that NRF2 is the strongest predictor of *SPP1* expression, followed by macrophage and CAF (cancer-associated fibroblast) cell infiltration. These data support the notion that deregulated *SPP1*, potentially as a central NRF2-regulated gene, contributes to tumor spread beyond lymph node capsules, a key indicator of poor prognosis in HNSCC. Collectively, these findings highlight a critical role for deregulated SPP1 in the locoregional and metastatic progression associated with cisplatin resistance in HNSCC. In HPV-positive OPSCC, higher SPP1 expression is paradoxically associated with improved prognosis. This association likely reflects SPP1 expression driven predominantly by tumor-associated macrophages rather than tumor cells, as indicated by the weaker contribution of the NRF2 signature and the stronger regression coefficients for macrophage-related scores. Consistent with this interpretation, HPV-positive tumors may lack KEAP1/NRF2 pathway alterations that promote higher intratumoral SPP1 expression, and survival outcomes instead dominated by HPV-associated tumor biology. Consequently, the relative contributions of intratumoral versus tumor immune microenvironment-derived SPP1 may differ substantially in HPV-positive disease, resulting in distinct prognostic implications.

In an in vitro competition assay, the use of anti-*SPP1* antibodies to block its interaction with cell surface receptors demonstrated that reduced tumor progression was specifically attributable to *SPP1* silencing. However, in vivo, treatment with a humanized anti-osteopontin (OPN) antibody alone had minimal effect on tumor growth. Notably, when combined with cisplatin, the antibody significantly inhibited tumor growth and metastasis in cisplatin-resistant models, consistent with prior findings [32]. These results suggest that *SPP1* inhibition may enhance cisplatin efficacy by potentially improving its ability to induce ferroptosis. Although anti-SPP1 treatment produced consistent effects in vivo, its combination with cisplatin in vitro yielded variable outcomes across experiments. These differences may be attributed to the multifaceted role of SPP1 in modulating the tumor microenvironment (TME) [40], highlighting the importance of in vivo contexts for fully capturing the therapeutic benefit of SPP1 targeting. The lack of effect from anti-OPN monotherapy suggests that while *SPP1* promotes tumor growth, its actions are not solely autonomous. The interaction of *SPP1* with two major receptor families, integrins and CD4 may activate distinct downstream signaling pathways [16–18]. This hypothesis is supported by co-immunoprecipitation and Visium spatial transcriptomics data showing *SPP1* interaction with both integrins and CD44 in HNSCC tumors. Inhibition of only one receptor-mediated pathway may therefore be insufficient to completely block *SPP1* signaling. Interestingly, we did not detect *SPP1*-CD44 interactions in vitro. However, spatial transcriptomics revealed their co-expression specifically in metastatic lung lesions of HNSCC patient tumors, suggesting a context-dependent role for this interaction within the tumor microenvironment (TME). These findings imply that targeting *SPP1*, particularly its interactions with integrin and CD44, could be a promising strategy to suppress tumor progression in NRF2-hyperactivated HNSCC.

Elevated plasma *SPP1* levels have been observed in head and neck cancer patients following surgery [41] and radiotherapy [42]. In light of these observations, future studies should explore whether baseline serum and saliva *SPP1* levels can predict treatment responses to cisplatin-radiation or immune checkpoint inhibitor (ICI) combination therapies in patients with advanced HNSCC. To assess the clinical utility of these findings, we successfully detected secreted *SPP1* in plasma samples from a small cohort of HNSCC patients (Supplementary Table S8). Through proteomic analysis, we examined the molecular mechanisms underlying *SPP1*-mediated tumor progression and cisplatin resistance. In line with prior studies [43–46], *SPP1* knockdown inhibited several key oncogenic, therapy resistance, and metastatic signaling pathways, including AKT/mTOR, PAK1 (p21-activated kinase 1), and FAK (focal adhesion kinase). To establish whether integrin/CD44 signaling axis is truly required for the observed SPP1-mediated effects, The observed reduction in FAK and PAK1 phosphorylation following *SPP1* silencing suggests a regulatory relationship between these pathways and *SPP1* and deserves further investigation.

Spatial transcriptomic and gene set enrichment analyses revealed differential *SPP1* expression across tumor clusters in HNSCC patients with distinct cancer hallmark pathway profiles. In primary tumors, *SPP1* levels were low and accompanied by EMT-related gene enrichment. In contrast, metastatic lung tumors showed high *SPP1* expression and enrichment of interferon response genes. These results suggest that *SPP1* may drive tumor growth via EMT regulation in early stages and facilitate metastasis through interactions within the metastatic TME. Supporting this, a recent study demonstrated that *SPP1* promotes cancer progression and chemoradiotherapy resistance through EMT, mediated by activation of the PI3K/Akt and MAPK pathways via binding to integrin αvβ3 and CD44 [15]. Our finding of *SPP1* and interferon gene co-expression in metastatic lung tumors also suggests a potential role in augmenting immunosuppressive inflammatory responses, thereby facilitating metastasis in NRF2-activated HNSCC. *SPP1* further contributes to metastasis by influencing macrophage behavior within the TME [47, 48]. Specifically, *SPP1*-positive macrophages have been linked to immune suppression, tumor progression, early lymph node metastasis, and enhanced angiogenesis. Thus, future research should explore how *SPP1* expression patterns may promote immune tolerance and inhibit immune activation in NRF2-hyperactivated HNSCC. A limitation of this study is that the spatial transcriptomic analyses were performed on a single patient sample; therefore, additional cohorts are required to validate the spatial integration of SPP1 with its cognate receptors and their interactions within the tumor microenvironment of cisplatin-resistant head and neck cancers.

In summary, our data show that specific inhibition of *SPP1* suppresses tumor progression in NRF2-hyperactivated, cisplatin-resistant HNSCC. Furthermore, *SPP1* may serve as a diagnostic and prognostic biomarker, as well as a therapeutic target. Given its frequent overexpression in the tumor microenvironment, particularly in tumor-associated macrophages and cancer-associated fibroblasts, further studies are necessary to delineate the contributions of *SPP1* from these cell types in the presence or absence of NRF2 activation. Finally, since alternative translation of *SPP1* produces both intracellular and secreted isoforms capable of activating integrin or CD44-mediated signaling, functional studies are warranted to determine the distinct roles of these isoforms in NRF2-hyperactivated HNSCC progression.

## Electronic Supplementary Material

Below is the link to the electronic supplementary material.

Supplementary Material 1

Supplementary Material 2

Supplementary Material 3

Supplementary Material 4

Supplementary Material 5

Supplementary Material 6

Supplementary Material 7

Supplementary Material 8

Supplementary Material 9

Supplementary Material 10

Supplementary Material 11

Supplementary Material 12

Supplementary Material 13

Supplementary Material 14

Supplementary Material 15

Supplementary Material 16

Supplementary Material 17

Supplementary Material 18

Supplementary Material 19

Supplementary Material 20

Supplementary Material 21

Supplementary Material 22

## Data Availability

The data generated in this study are available within the article and its supplementary files.

## Abbreviations

HNSCC: Head and neck squamous cell carcinoma
CDDP: Cisplatin
KEAP1: Kelch-like ECH-associated protein 1
NRF2: Nuclear factor erythroid 2–related factor 2
CUL3: Cullin 3
OCSCC: Oral cavity squamous cell carcinoma
SPP1: Secreted phosphoprotein 1
OPN: Osteopontin
GPX4: Glutathione peroxidase 4
ACSL4: Acyl-CoA synthetase long chain family member 4
