# Supplementary figures and images for: Targeted suppression of SPP1 inhibits tumor invasion and metastasis in NRF2 hyperactivated cisplatin resistant HNSCC
Source: J Transl Med. 2026 May 22;24:926. doi: 10.1186/s12967-026-08292-x (PMC13383472; doi:10.1186/s12967-026-08292-x)

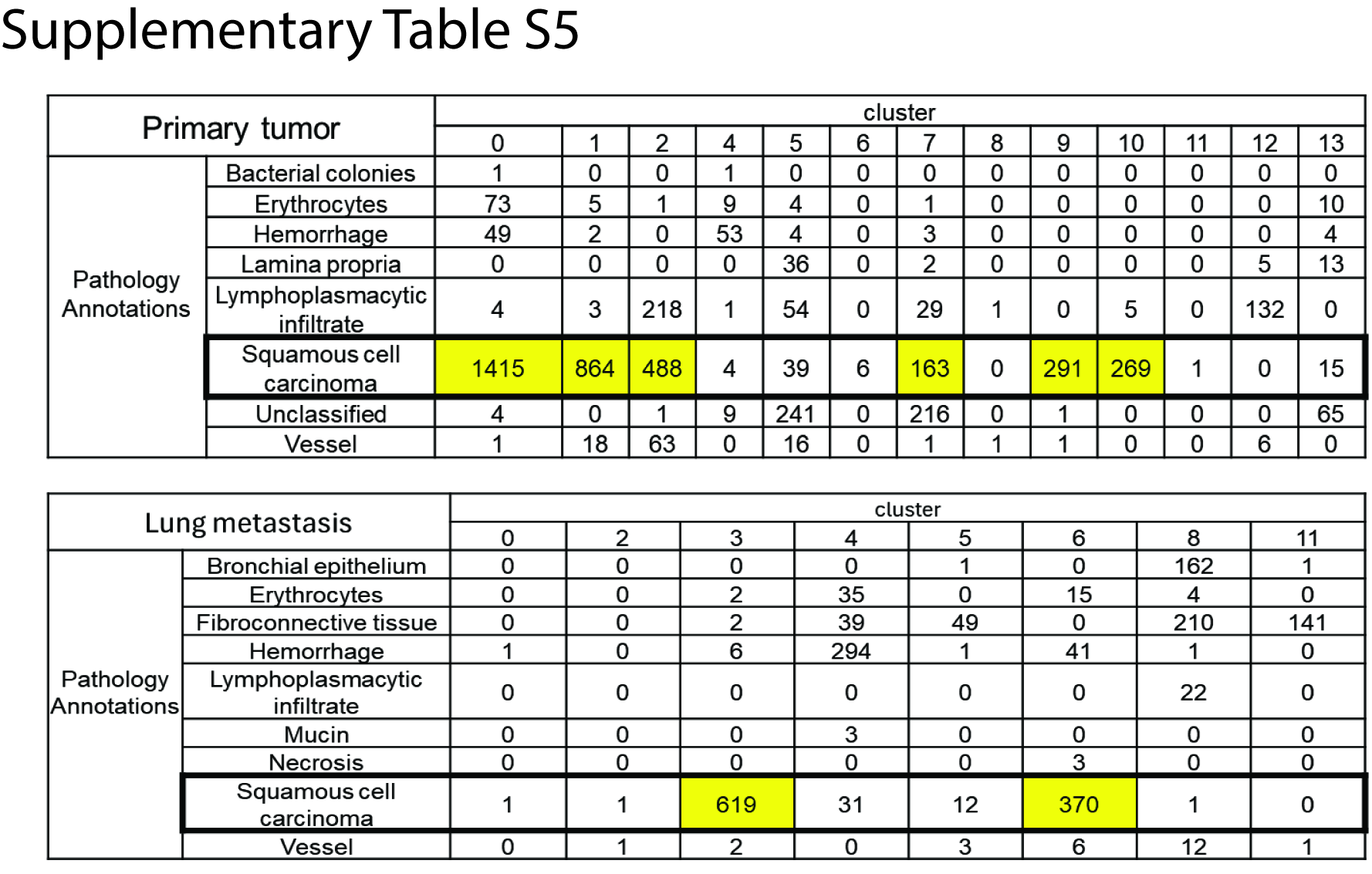

Supplement: Supplementary file 8 — Supplementary Material 8 [file 12967_2026_8292_MOESM8_ESM.tif]

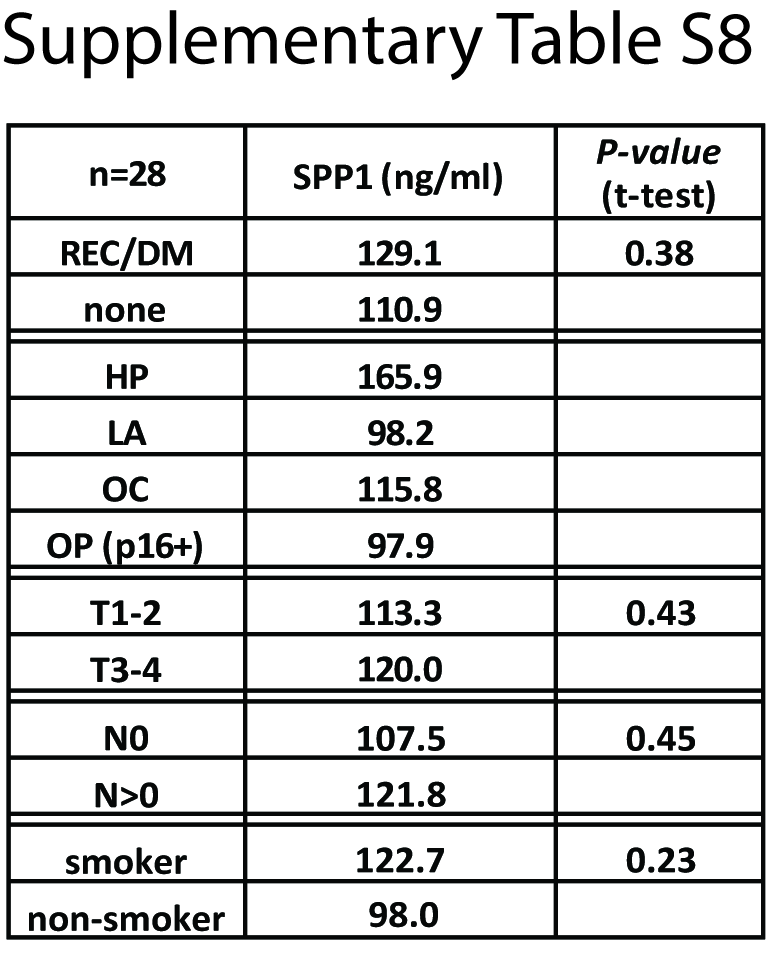

Supplement: Supplementary file 9 — Supplementary Material 9 [file 12967_2026_8292_MOESM9_ESM.tif]

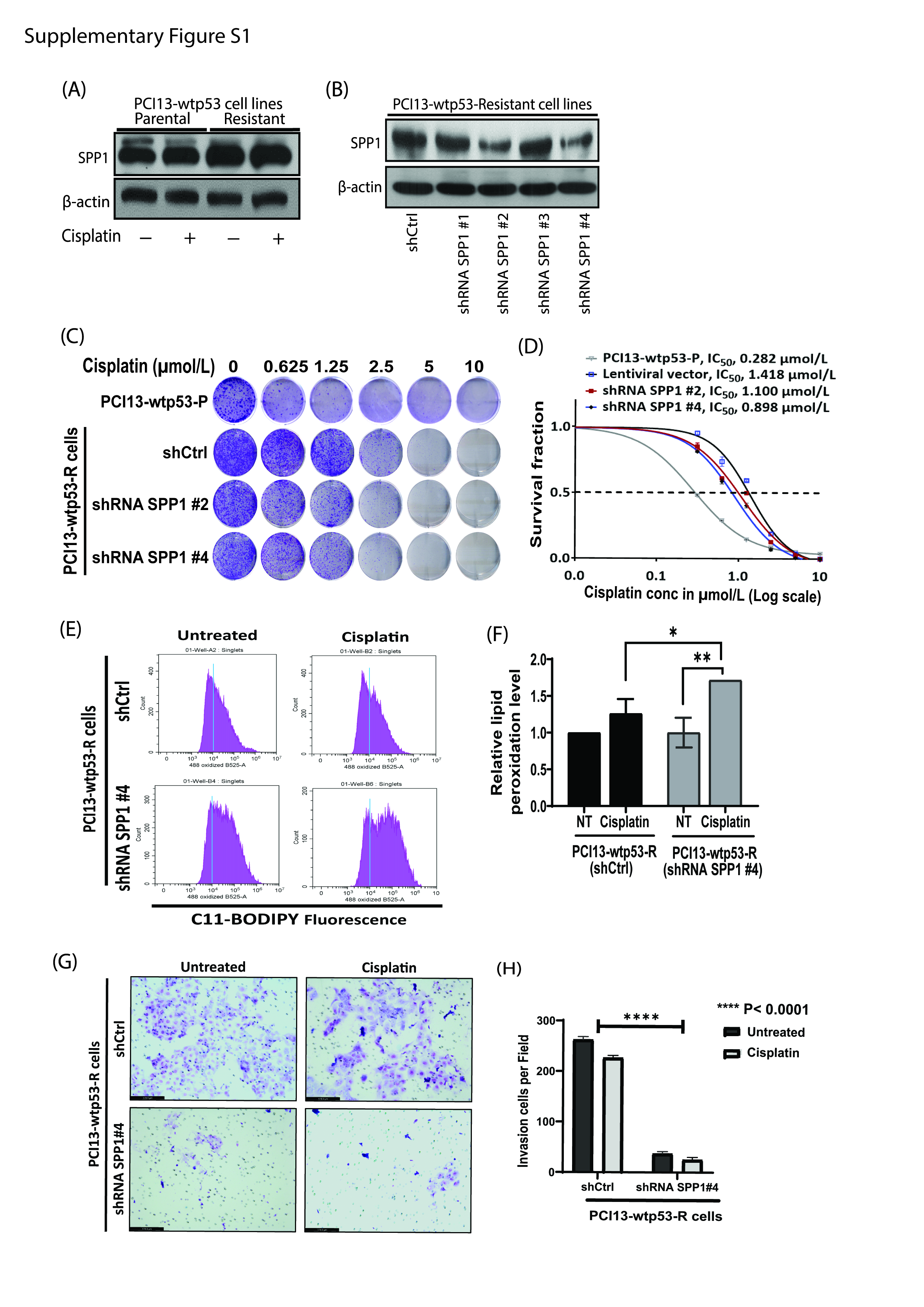

Supplement: Supplementary file 11 — Supplementary Material 11 [file 12967_2026_8292_MOESM11_ESM.tif]

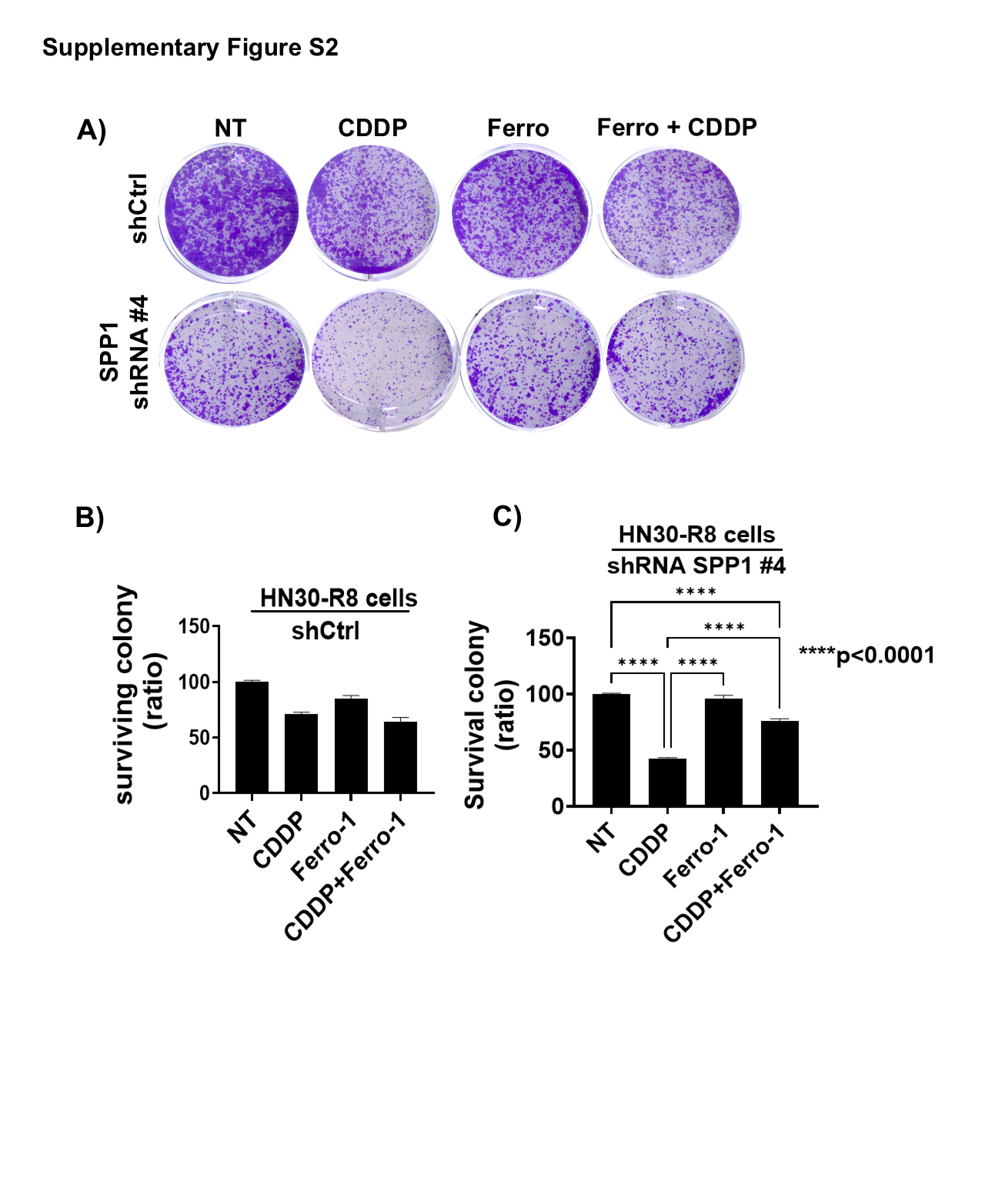

Supplement: Supplementary file 12 — Supplementary Material 12 [file 12967_2026_8292_MOESM12_ESM.tif]

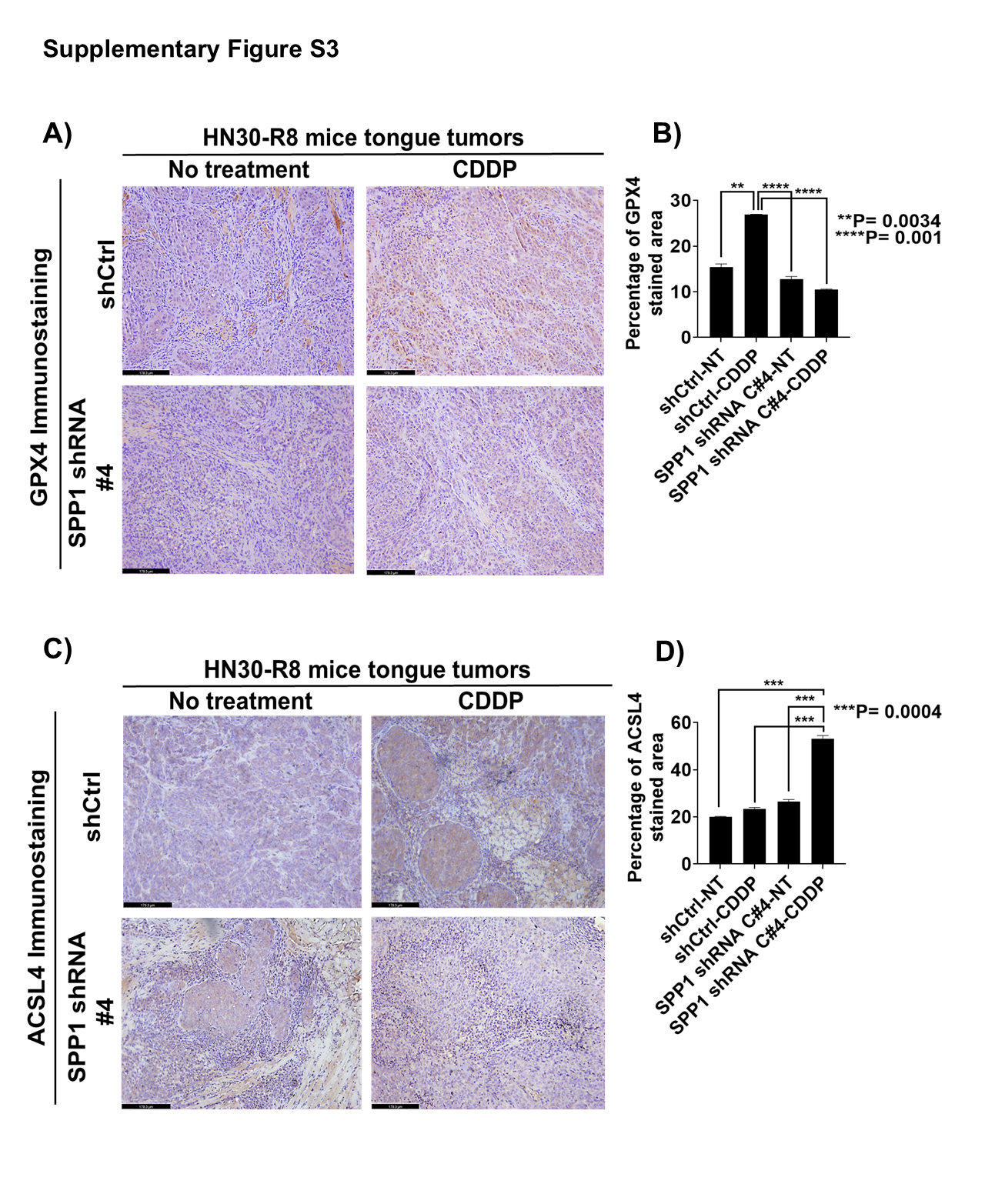

Supplement: Supplementary file 13 — Supplementary Material 13 [file 12967_2026_8292_MOESM13_ESM.tif]

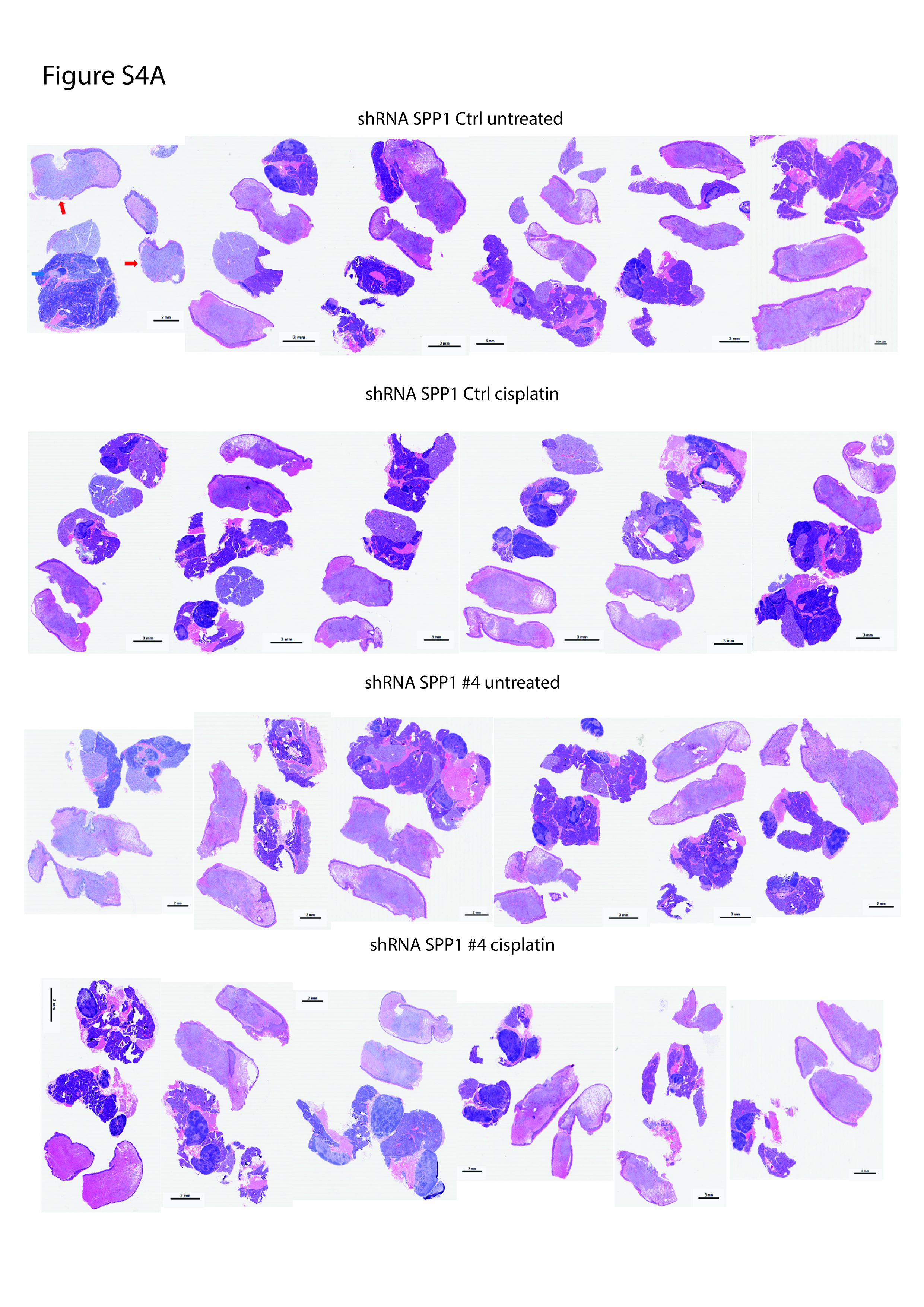

Supplement: Supplementary file 14 — Supplementary Material 14 [file 12967_2026_8292_MOESM14_ESM.tif]

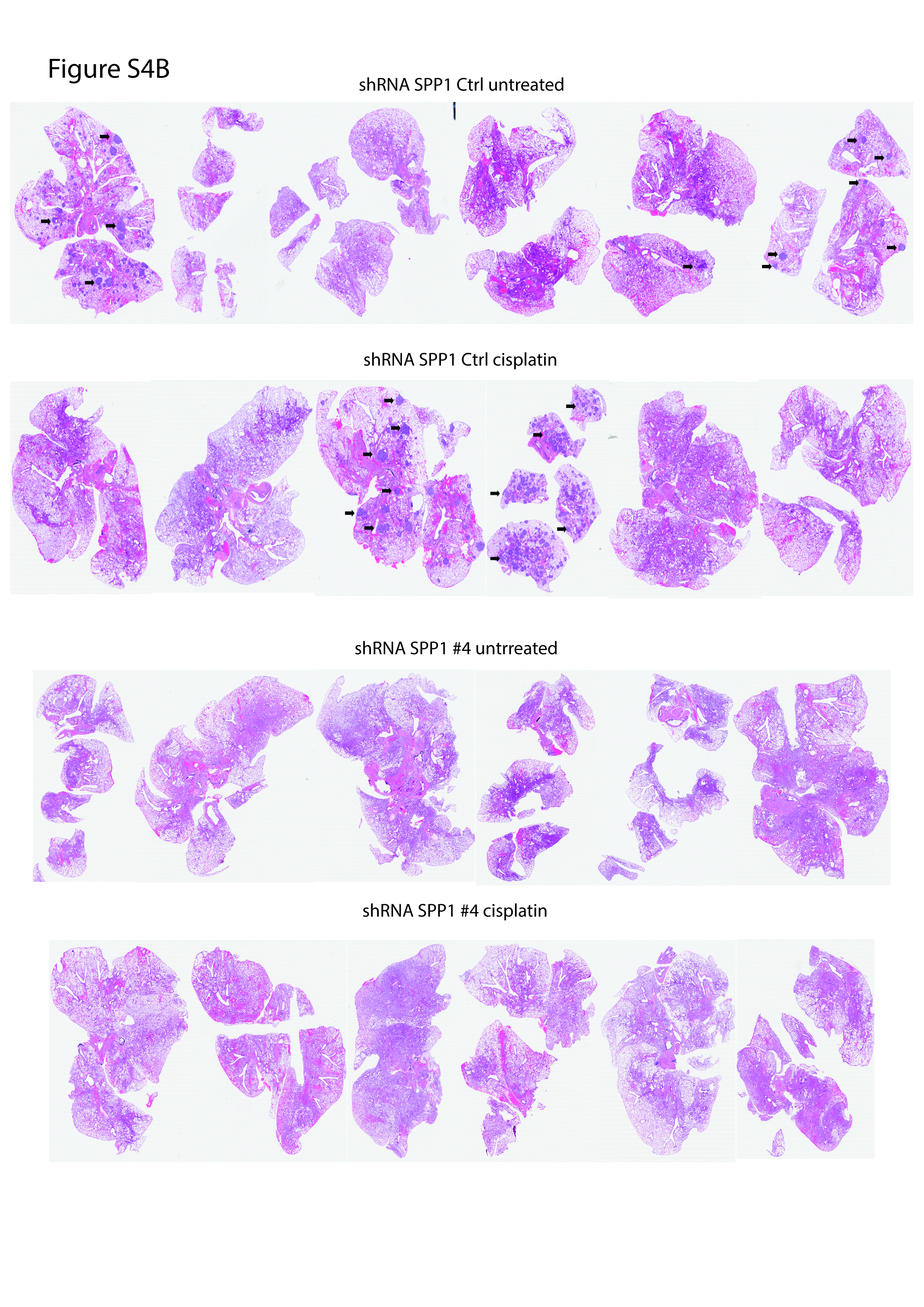

Supplement: Supplementary file 15 — Supplementary Material 15 [file 12967_2026_8292_MOESM15_ESM.tif]

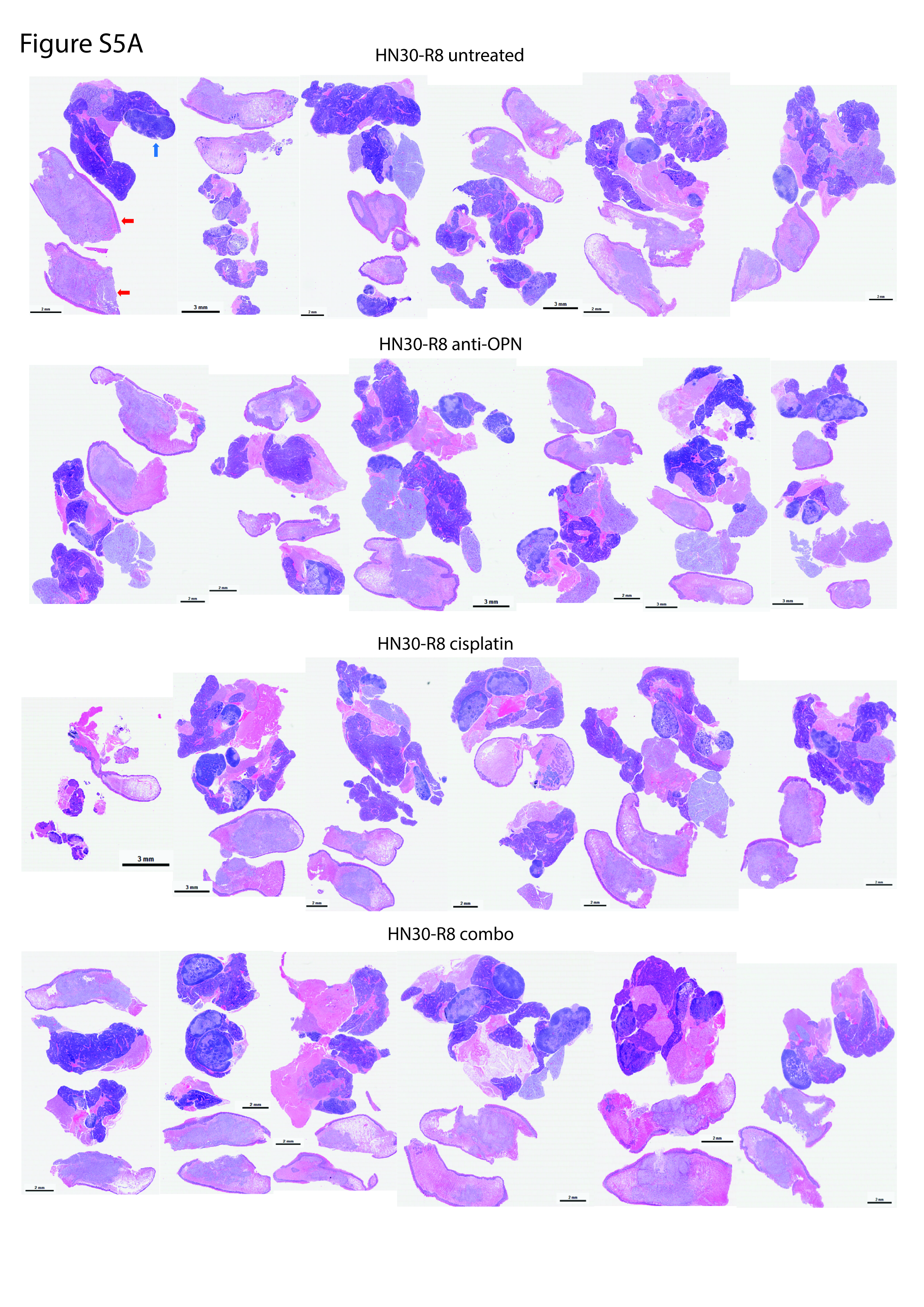

Supplement: Supplementary file 16 — Supplementary Material 16 [file 12967_2026_8292_MOESM16_ESM.tif]

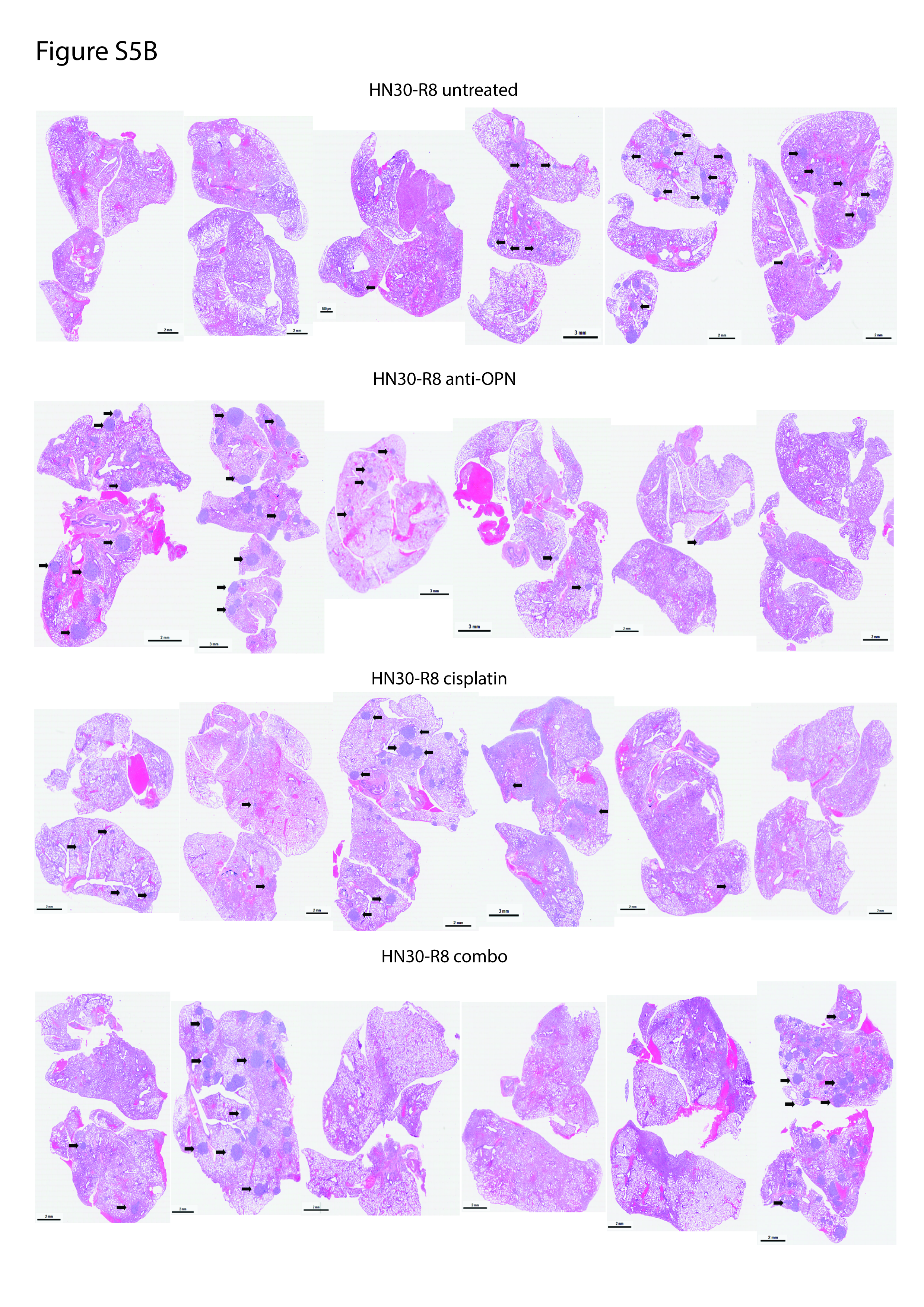

Supplement: Supplementary file 17 — Supplementary Material 17 [file 12967_2026_8292_MOESM17_ESM.tif]

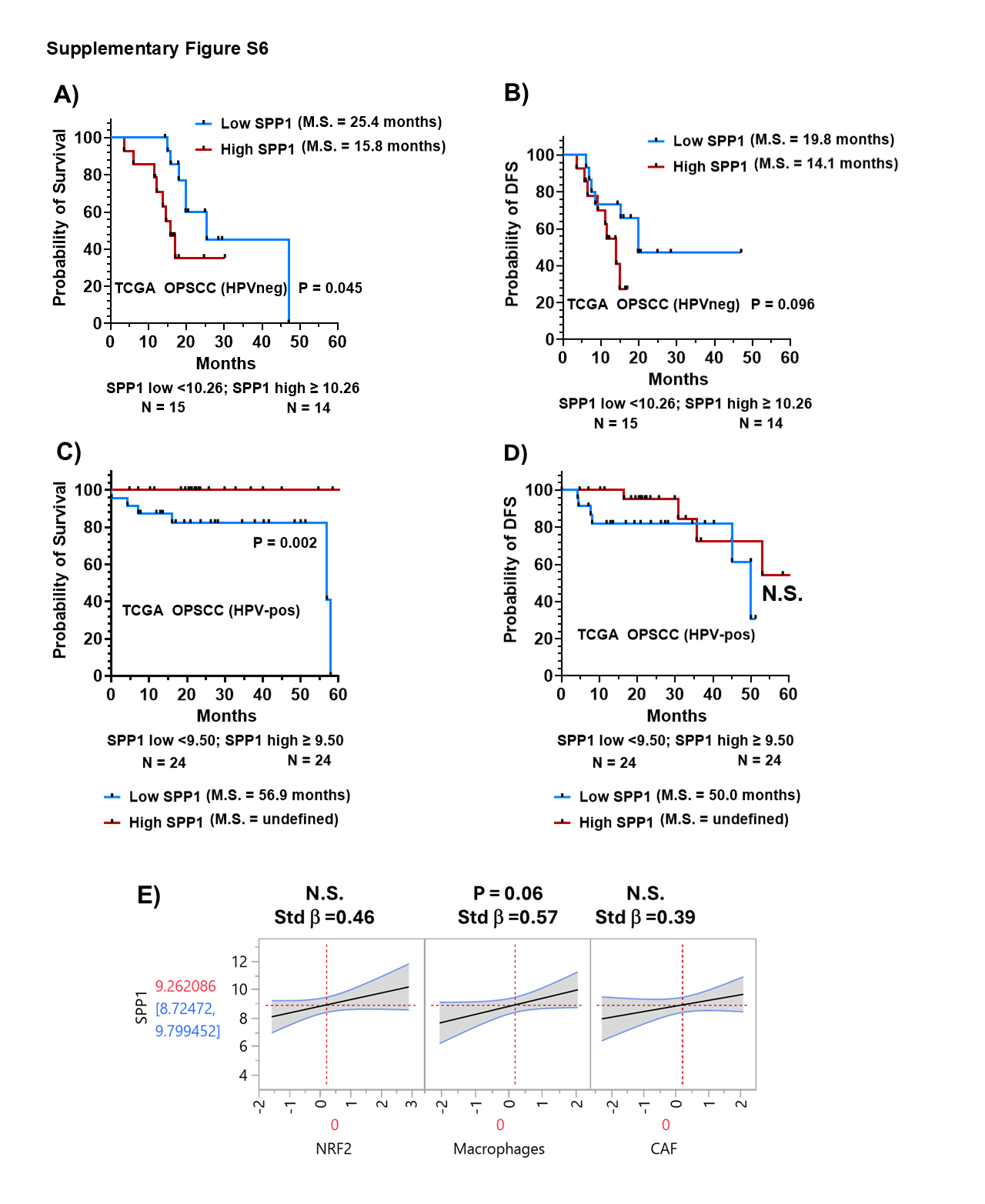

Supplement: Supplementary file 18 — Supplementary Material 18 [file 12967_2026_8292_MOESM18_ESM.tif]

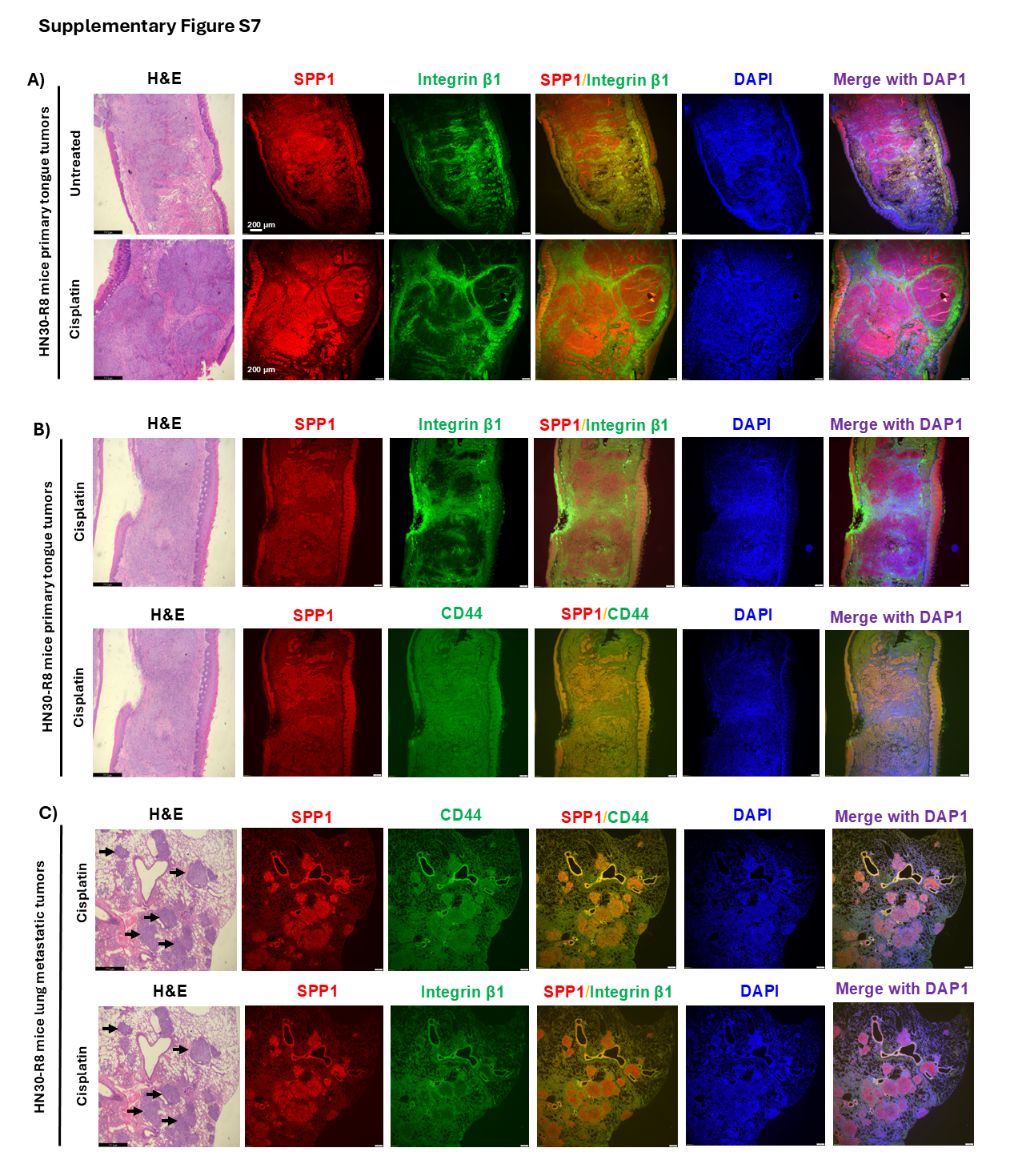

Supplement: Supplementary file 19 — Supplementary Material 19 [file 12967_2026_8292_MOESM19_ESM.tif]

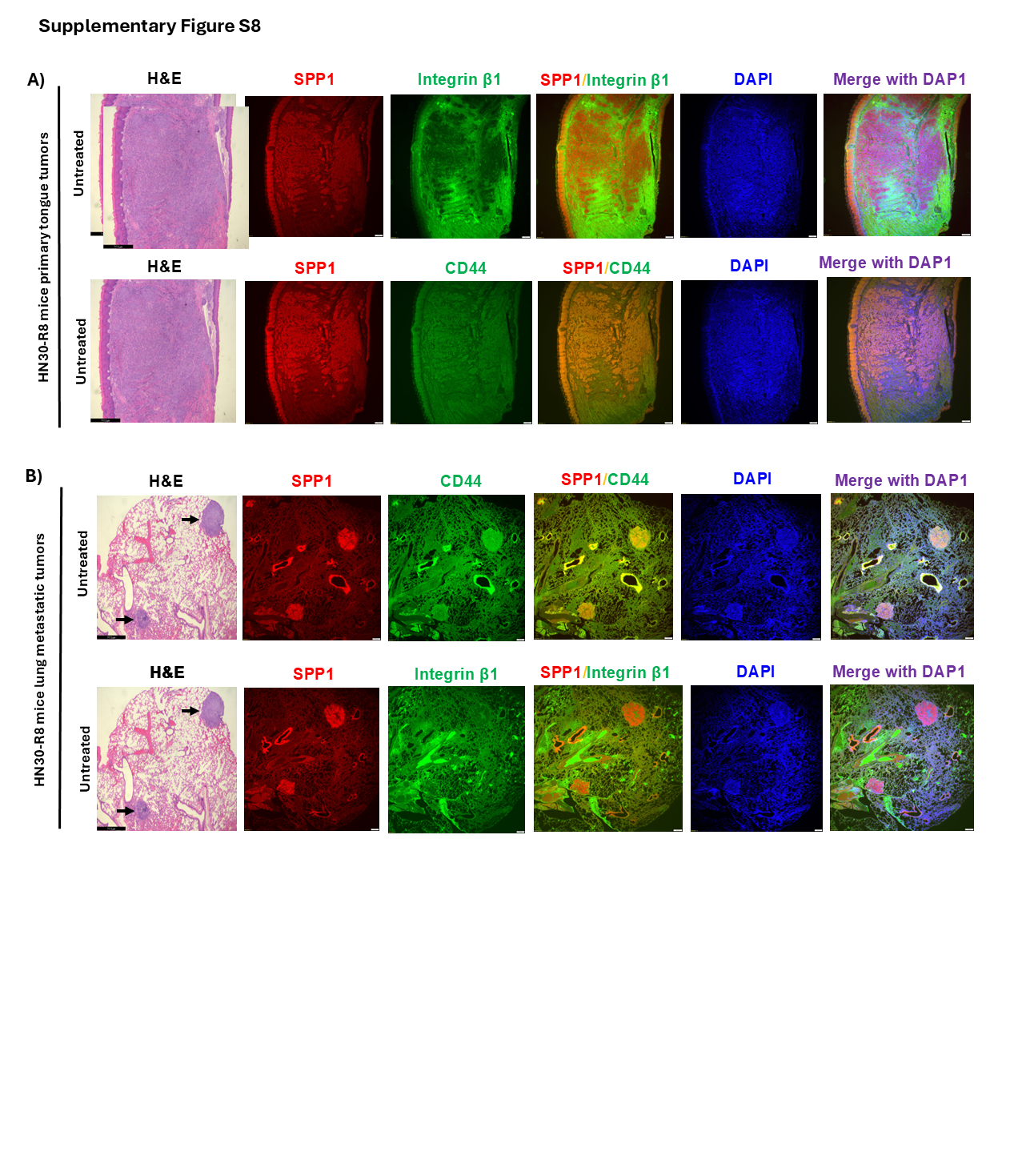

Supplement: Supplementary file 20 — Supplementary Material 20 [file 12967_2026_8292_MOESM20_ESM.tif]

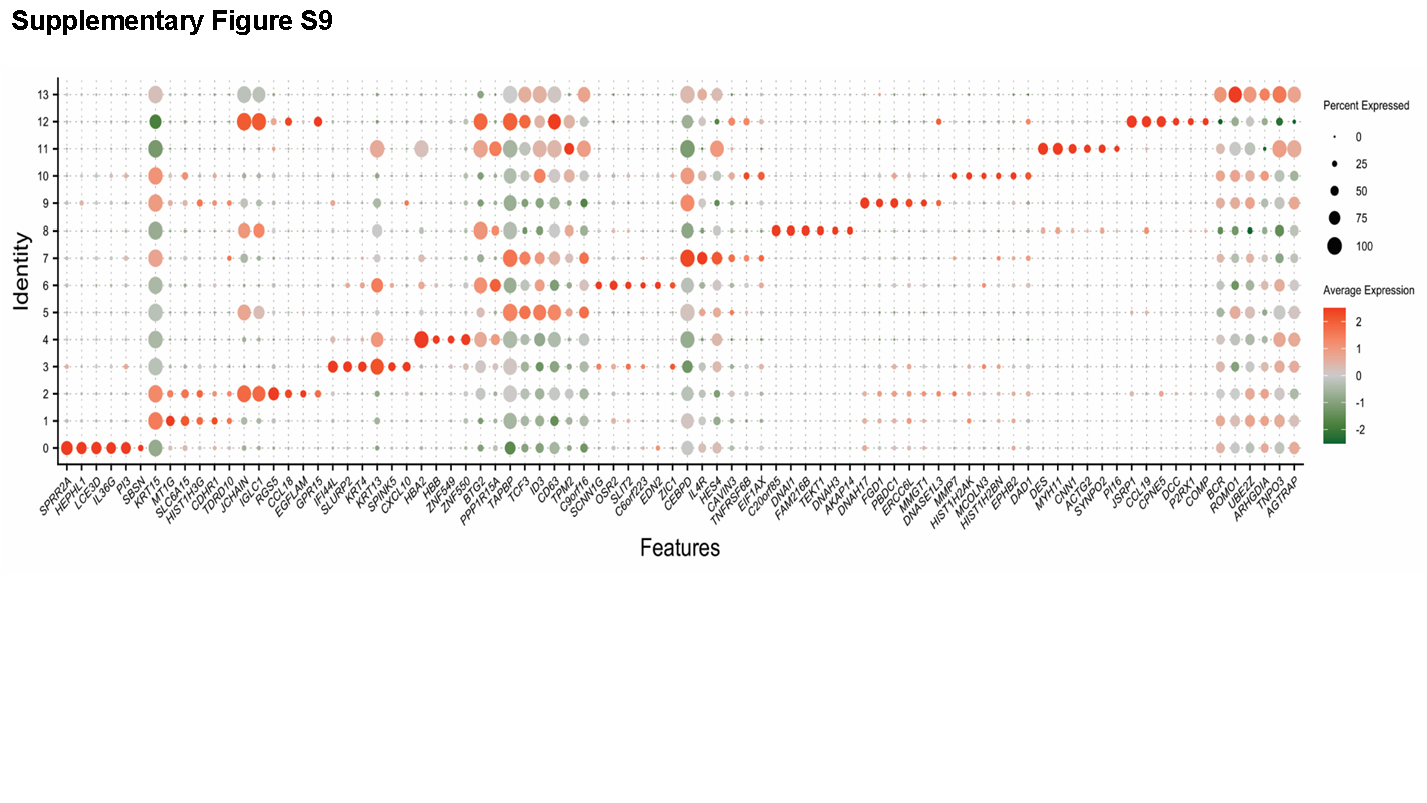

Supplement: Supplementary file 21 — Supplementary Material 21 [file 12967_2026_8292_MOESM21_ESM.tif]
