# Supplementary material for: Targeted suppression of SPP1 inhibits tumor invasion and metastasis in NRF2 hyperactivated cisplatin resistant HNSCC
Source: J Transl Med. 2026 May 22;24:926. doi: 10.1186/s12967-026-08292-x (PMC13383472; doi:10.1186/s12967-026-08292-x)
Supplement: Supplementary file 10 — Supplementary Material 10 [file 12967_2026_8292_MOESM10_ESM.docx]

**Targeted suppression of SPP1 inhibits tumor invasion and metastasis in Nrf2 hyperactivated cisplatin resistant HNSCC**

Kawabe M et al.

**Supplementary Materials and Methods**

**Immunohistochemistry**

For detection of ferroptosis and oxidative stress markers, sections were prepared from formalin-fixed paraffin embedded mice tumor tissues (N =2). The sections were incubated with 3% H2O2 for 10 minutes to block endogenous peroxidase activity. The antigen was retrieved by incubation at 95 °C in citric acid antigen repair solution for 30 minutes. Following blocking in methanol and water, sections were incubated in 2.5% normal goat serum made in TBST for 30 minutes, washed and stained with anti-GPX4 mouse mAb (1:100, Cat# (SC-166570), Santa Cruz Biotechnology, Inc), anti-ACSL4mouse mAb (1:100, Cat #Sc-271800, Santa Cruz Biotechnology, Inc), at 4 °C overnight. Binding antibodies were detected using conjugated secondary antibodies as appropriate and incubated for 1 h at room temperature. The sections were then developed with DAB solution, and counterstained with hematoxylin. Images were acquired using Leica DMLA microscope (Leica Microsystems) and captured at 179.3 µm scale bar. Immunohistochemical images were quantified using ImageJ software (NIH) and analyzed with GraphPad Prism 6 (GraphPad Software, San Diego, CA).The quantification was defined as the density of cells and average optical density per area (n = 3 stained tumors; every antibody staining had 5 field views).

**Immunofluorescence**

For co-localization experiments, mouse tumor sections (N = 2) were deparaffinized in three changes of xylene for 5 min each and rehydrated through a graded ethanol series (100%, 90%, and 80%) for 5 min each. Slides were then rinsed three times in deionized water for 5 min and subjected to antigen retrieval by boiling at 90 °C for 40 min, followed by additional rinses in deionized water. Tissue sections were permeabilized with 0.5% Triton X-100 for 15 min, washed, and blocked with 3% bovine serum albumin (BSA) for 30 min. Slides were subsequently incubated overnight at 4 °C with the following primary antibodies: SPP1 (Proteintech, Cat# 22952-1-AP; 1:200), Integrin β1 (Santa Cruz Biotechnology, Cat# sc-374429; 1:200), and CD44 monoclonal antibody (IM7; eBioscience™, Cat# 14-0441-82, Invitrogen; 1:200). After washing, sections were incubated for 1 h at room temperature with the appropriate secondary antibodies: goat anti-rabbit IgG (H+L), Alexa Fluor™ 594 (Invitrogen, Cat# A-11037; 1:400); goat anti-mouse IgG (H+L), Alexa Fluor™ 488 (Invitrogen, Cat# A-11029; 1:400); or goat anti-rat IgG (H+L), Alexa Fluor™ 488 (Invitrogen, Cat# A-11006; 1:400), along with DAPI (Thermo Scientific™, Cat# 62248; 1:1000). Following washing, immunofluorescence images were acquired using an Olympus IX71 confocal microscope and captured with a 200 µm scale bar.
