# Supplementary material for: Targeted suppression of SPP1 inhibits tumor invasion and metastasis in NRF2 hyperactivated cisplatin resistant HNSCC
Source: J Transl Med. 2026 May 22;24:926. doi: 10.1186/s12967-026-08292-x (PMC13383472; doi:10.1186/s12967-026-08292-x)
Supplement: Supplementary file 22 — Supplementary Material 22 [file 12967_2026_8292_MOESM22_ESM.docx]

**Targeted suppression of SPP1 inhibits tumor invasion and metastasis in Nrf2 hyperactivated cisplatin resistant HNSCC**

*Kawabe M et al.*

**Supplementary Figure and Table Legends**

**Supplementary Figure S1. Downregulation of SPP1 enhances cisplatin sensitivity *in vitro* in cisplatin resistant HNSCC through induction of ferroptosis.** **A,** Western blot demonstrates high expression of SPP1 during acquired CDDP resistance in PCI13-wtp53 HNSCC cells compared to PCI13-wtp53 CDDP sensitive parental cells. **B,** Western blot verifies downregulation efficiency of SPP1 in stable CDDP resistant cells (PCI13-wtp53-resistant). **C and D,** Representative images of clonogenic survival and curves demonstrating increased sensitivity to CDDP in PCI13-wtp53 cells stably expressing shRNA SPP1 after treatment with the indicated doses of cisplatin. The PCI13-wtp53-P, PCI13-wtp53-R-shCtrl and their shRNA SPP1 derivatives were cultured in presence and absence of CDDP (2.0 µmol/L) for 48 hr and subjected to Matrigel invasion and ferroptosis assays as described in Methods. P****<0.0001; shRNA SPP1 versus shCtrl control cells. **E,** Fluorescence level of intracellular oxidized C11-BODIPY (581/591) measured by Flow cytometry in the cells. **F,** Bar graph showing increased lipid peroxidation levels calculated from the fluorescent integrated density of the oxidized BODIPY indicates ferroptosis in PCI13-wtp53-R cells expressing SPP1 shRNA following treatment with CDDP compared to untreated ShCtrl control cells. **G and H,** Matrigel invasion images and bar graph demonstrating that the invasion ability of PCI13-wtp53-R cells expressing shRNA SPP1 is significantly decreased compared to the shCtrl control cells. Bar graphs are mean ± SEM, unpaired student t-test and two-way ANOVA, respectively. Experiments were performed in triplicates and repeated two independent times.

**Supplementary Figure S2. Blocking ferroptosis attenuates cisplatin-induced cell death in SPP1-knockdown cisplatin-resistant HNSCC cells *in vitro*.** To confirm specific cell death through ferroptosis, the cells were treated with various 5 µµmol/L of cisplatin (CDDP) and supplemented with ferrostatin-1 (1 µmol/L) and subjected to clonogenic survival assay as indicated in methods. A, Representative images of clonogenic survival assay. B and C, Bar graphs demonstrating decreased surviving colonies to CDDP in HN30-R8 cells stably expressing shRNA SPP1 #4 compared to shCtrl control cells following treatment with the ferroptosis inhibitor, Ferro-1 (Ferrostatin-1). P****<0.0001; CDDP + Ferro-1 versus CDDP treated shRNA SPP1cells.

**Supplementary Figure S3. SPP1 knockdown modulates oxidative stress and ferroptosis-associated markers in mouse tongue tumors *in vivo*.** Tongue tumor sections were collected from mice injected orally with cisplatin-resistant HN30-R8 cells expressing either shCtrl or SPP1 shRNA #4. Tumors were subjected to immunohistochemical analysis as described in the Methods. **A and B,** Representative immunohistochemistry images and corresponding quantification show reduced expression of GPX4, a ferroptosis inhibitor, in cisplatin (CDDP)–treated tumors with SPP1 knockdown compared with CDDP-treated shCtrl tumors. p = 0.0034, CDDP-treated versus untreated shCtrl groups; ****p = 0.001, CDDP-treated SPP1 shRNA versus CDDP-treated shCtrl groups. **C and D,** Representative immunohistochemistry images and corresponding quantification demonstrate increased expression of ACSL4, a ferroptosis activator, in CDDP-treated tumors with SPP1 knockdown compared with untreated and control treatment groups. ****p = 0.001, CDDP-treated SPP1 shRNA versus all other groups. Bar graphs are mean ± SEM, two-way ANOVA. N = 3 stained mice tumors. Scale bars: 179.3 µm.

**Supplementary Figure S4A. Tongue and lymph node harvested from the mice injected with HN30-R8 cells stably expressing shRNA Ctrl and shRNA SPP1 following cisplatin treatment.** Red arrows indicate tongue tumor, and blue arrows indicate cervical lymph node.

**Supplementary Figure S4B. Lungs harvested from the mice injected with HN30-R8 cells stably expressing shRNA Ctrl and shRNA SPP1 following cisplatin treatment.** Black arrows indicate metastatic lung tumor nodules.

**Supplementary Figure S5A. Tongue and lymph node harvested from the mice injected with HN30-R8 cells following treatment with cisplatin in combination with anti-SPP1 (anti-osteopontin).** Red arrows indicate tongue tumor, and blue arrows indicate cervical lymph node.

**Supplementary Figure S5B. Lungs harvested from the mice injected with HN30-R8 cells following treatment with cisplatin in combination with anti-SPP1 (anti-osteopontin).** Black arrows indicate metastatic lung tumor nodules.

**Supplementary Figure S6. SPP1 expression is associated with better survival in HPV-positive OPSCC**. The optimal SPP1 expression cutoff in the TCGA OPSCC cohort was determined using recursive partitioning. **A and B,** Kaplan–Meier analyses of overall survival and disease-free survival in HPV-negative oropharyngeal squamous cell carcinoma (OPSCC) patients stratified by SPP1 expression. **C and D,** Kaplan–Meier analyses of overall survival and disease-free survival in HPV-positive OPSCC patients stratified by SPP1 expression. HPV-positive tumors with elevated SPP1 mRNA expression exhibit a significantly longer median overall survival. **E,** Regression model analysis indicating that macrophages have relatively stronger regression coefficient and NRF2 and cancer-associated fibroblasts (CAFs) are weak predictors of SPP1 expression.

**Supplementary Figure S7. Co-localization of SPP1, integrin and CD44 in vivo in mice tongue and lung tumors bearing cisplatin resistant HNSCC cells.** Tongue tumor sections were collected from mice injected orally with cisplatin-resistant HN30-R8 cells and subjected to immunofluorescence analysis as described in the Methods. **A and B,** Representative confocal immunofluorescence images of CDDP treated and untreated primary tongue tumors stained with SPP1 (red), integrin β1 (green), CD44 antibodies and DAPI (blue). Intense co-localization of SPP1 with integrin β1 and CD44 was observed in the primary tumors treated with cisplatin (double staining, orange ;and triple staining, purple). **C,** Representative confocal immunofluorescence images of CDDP treated and untreated lung metastatic tumors stained with SPP1 (red), CD44 (green) , integrin β1 (green) antibodies and DAPI (blue). Intense co-localization of SPP1 with CD44 and integrin β1 was observed in the primary tumors treated with cisplatin (double staining, orange ;and triple staining, purple). Tumors with corresponding H&E staining are shown. N = 2 stained mice tumors. Scale bars: 717.7 µm for the H&E, and 200 µm for the immunestained images, respectively.

**Supplementary Figure S8. Co-localization of SPP1, integrin and CD44 in vivo in mice tongue and lung tumors bearing cisplatin resistant HNSCC cells.** Tongue tumor sections were collected from mice injected orally with cisplatin-resistant HN30-R8 cells and subjected to immunofluorescence analysis as described in the Methods. **A and B,** Representative confocal immunofluorescence images of CDDP untreated primary tongue tumors stained with SPP1 (red), integrin β1 (green), CD44 antibodies and DAPI (blue). Intense co-localization of SPP1 with integrin β1 and CD44 was observed in the primary tumors (double staining, orange ;and triple staining, purple). Tumors with corresponding H&E staining are shown. N = 2 stained mice tumors. Scale bars: 717.7 µm for the H&E, and 200 µm for the immunestained images, respectively.

**Supplementary Figure S9.** Bubble plot annotating identified cell types and clusters expressing the top 6 significantly expressed genes in each cluster.

**Supplementary Table S5. Pathological annotation of the clusters in primary and metastatic lung tumors analyzed by Seurat.** The tabulated annotation shows the population of cell types in each cluster present in the primary and metastatic lung tumors. Clusters with higher number of squamous cell carcinoma type were selected for analysis.

**Supplementary Table S8. ELISA analysis of SPP1 (osteopontin) in serum obtained from HNSCC patients.** Blood samples were obtained from advanced HNSCC patients processed and subjected to ELISA analysis as described in Methods according to manufacturer’s procedures. The ELISA kit is designed to detect human OPN in serum with a detection limit ≥ 2.2 ng/ml. Results showed that SPP1 levels were slightly elevated in the serum from these patients and correlated with tumor grade and smoking but did not reach significance
